# Thermogravimetric analysis of commercial tungsten molecular precursors for vapor phase deposition processes†

**DOI:** 10.1039/d4ra07284g

**Published:** 2024-12-19

**Authors:** Taylor M. Currie, Terrick McNealy-James, S. Novia Berriel, Konstantin Preradovic, Alfred P. Sattelberger, Parag Banerjee, Titel Jurca

**Affiliations:** a Department of Chemistry, University of Central Florida Orlando Florida 32816 USA titel.jurca@ucf.edu; b Renewable Energy and Chemical Transformations Cluster, University of Central Florida Orlando Florida 32816 USA; c Department of Materials Science and Engineering, University of Central Florida Orlando Florida 32816 USA; d NanoScience & Technology Center, University of Central Florida Orlando Florida 32826 USA; e Florida Solar Energy Center, University of Central Florida Cocoa Florida 32922 USA

## Abstract

Thin films and coatings based on Group 6 metal tungsten (W) have garnered intense interest for applications including catalysis, lubrication, and solar energy. Due to its selectivity and conformality, atomic layer deposition (ALD) has emerged as a key route towards oxides, dichalcogenides, and elemental metal films of W. A key component of the ALD process is the appropriate selection of molecular precursors. Thermogravimetric analysis (TGA) is the primary sorting criterion for precursor suitability and helps delineate probable ALD temperature windows, sublimation/vaporization kinetics, and parameters of decomposition. Currently, a majority of the W materials growth literature is traced to a grouping of commercially available volatile molecular precursors. However, no comprehensive thermochemical analysis exists for all of these precursors, hampering a meaningful direct comparison. Herein, we present an extensive thermogravimetric analysis and direct comparison of commercial volatile W molecular precursors. We report probable ALD temperature windows, enthalpies of sublimation (Δ*H*_sub_), activation energies (*E*_a_), and evaluate thermal stress. Our findings highlight several commercial precursors yet to be reported for ALD growth, but featuring thermochemical properties falling within our suitable observed parametric ranges indicative of potential/viable deposition processes.

## Abstract 2

In depth thermogravimetric analysis and direct comparison of commercial volatile molecular tungsten-based precursors for atomic layer deposition.

## Graphical abstract

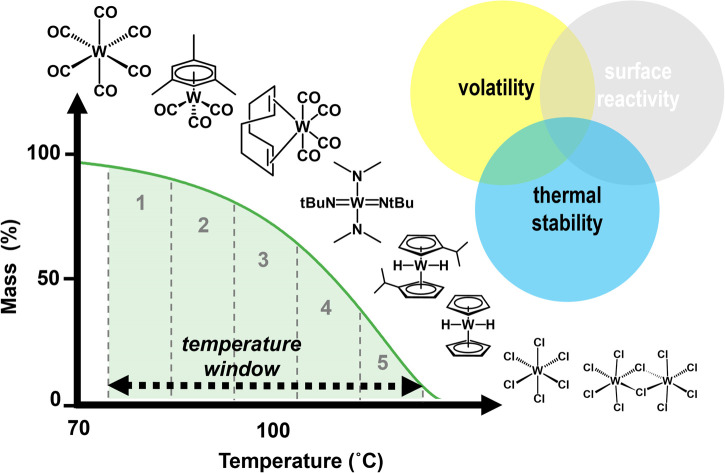

## 1. Introduction

Due to broad ranging applications in electronics, sensing, solar energy materials, lubricants and catalysis, thin films based on the Group 6 metal tungsten (W) have garnered intense interest in recent years.^1–7^ The majority of the focus has been on the oxides WO_*x*_ (2 ≤ *x* ≤ 3) and dichalcogenides WE_2_ (E = S, Se, Te), and to a lesser extent elemental metal, metal nitride, and metal carbide films. While chemical vapor deposition (CVD) has been the traditional preparative route, limitations in thickness control and conformality over larger domains has ushered in the development of atomic layer deposition (ALD)-based routes for these materials. Concomitantly, this has generated interest in the development of suitable W-based molecular precursors to enable film growth. As ALD precursors are often highly reactive and air- and moisture-sensitive, requiring significant background in molecular inorganic synthesis, most end-users are heavily reliant on a limited group of commercial precursors.

**Fig. 1:**
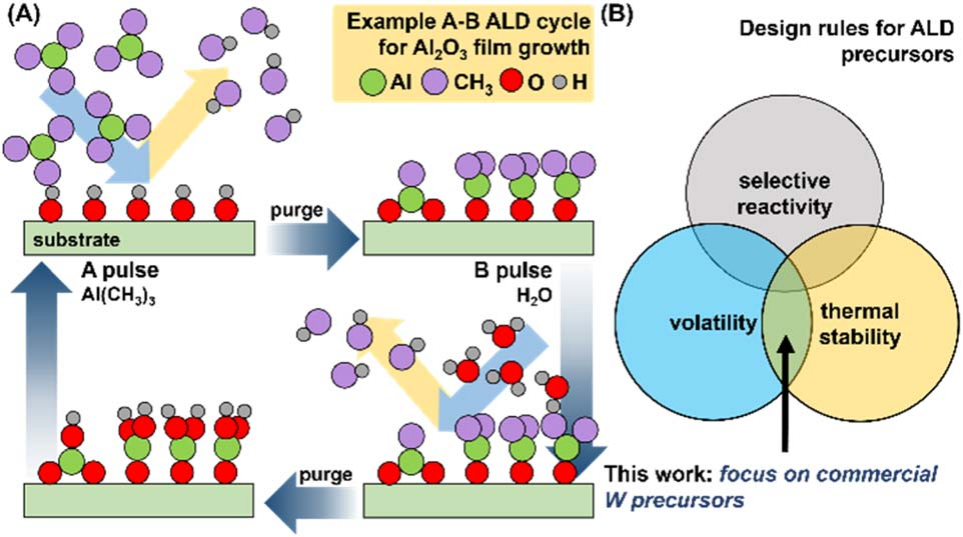
(A) Schematic representation of growth sequence for Al_2_O_3_ film by ALD using Al(CH_3_)_3_ and H_2_O in an A–B cycle; an “ideal” ALD process. (B) Venn diagram of desirable characteristics for viable ALD precursors.

ALD is a vapor-phase process that utilizes alternating pulses of volatilized chemical precursors containing the elemental components of interest in the growing film (typically a metal, and an oxidant or reductant).^8,9^ An example of the growth of Al_2_O_3_ with molecular precursors Al(CH_3_)_3_ and H_2_O in an A–B cycle, *one of the best understood ALD processes*, is highlighted in Fig. 1A.

Optimal process parameters for ALD growth (temperature, pulse duration, number of cycles) are dependent on the physicochemical properties of both the precursors and the substrate. ALD precursor viability is predicated on three key attributes: (i) volatility, (ii) thermal stability, and (iii) selective surface reactivity (Fig. 1B).^10,11^ Testing all three attributes naturally requires the use of an ALD reactor, and multi-parametric optimization (*vide supra*) and often significant amounts of precursors (gram scale). However, volatility and thermal stability are the primary criteria before surface reactivity can even be evaluated. Thermogravimetric analysis (TGA) only requires milligram amounts of analyte and can rapidly confirm both volatility and thermal stability of molecular precursors. This can facilitate rapid down-selection of precursor without the need for immediate synthetic scale-up and valuable ALD reactor time. Comprehensive multi-parameter screening can also provide operational TGA “temperature windows”, and related sublimation parameters such as enthalpy of sublimation (Δ*H*_sub_), activation energy (*E*_a_), and 1 Torr temperatures; all valuable metrics which can facilitate comparisons between larger pools of precursors and inform operational parameters for ALD growth of thin films. With these values in hand, researchers can select the appropriate precursor(s) for their targeted ALD processes.

**Scheme 1:**
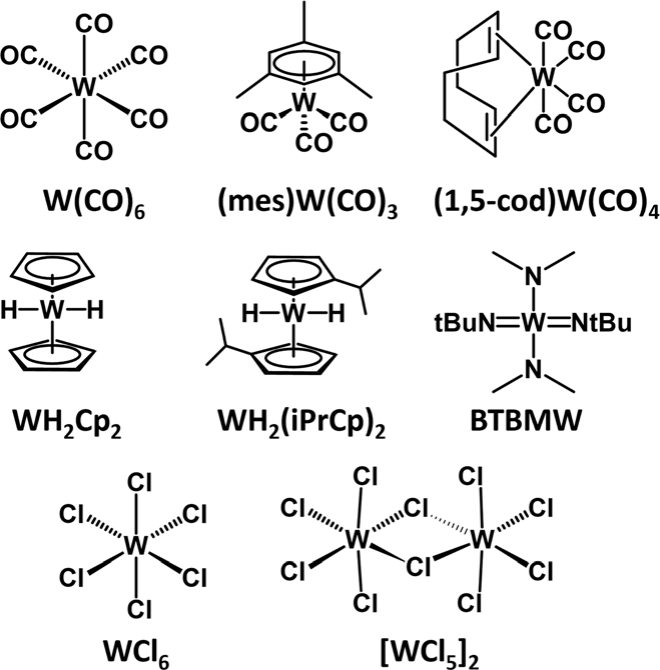
Commercially available W-based precursors subjected to non-isothermal TGA in this report.

In this paper, we report the thermochemical properties of a group of commercial W precursors (Scheme 1) evaluated using TGA. The chosen molecules can be broadly categorized according to their general ligand classes; carbonyls [W(CO)_6_, (1,5-cod)W(CO)_4_], cyclopentadienyl [WH_2_Cp_2_, WH_2_(iPrCp)_2_], chlorides [[WCl_5_]_2_, WCl_6_], and amino/imido [BTBMW]. While the reporting of thermochemical parameters for these precursors has been treated with varying degrees of rigor spanning multiple publications over several decades, a systematic and comparative investigation has been lacking. Compounded with the fact that TGA-obtained data is dependent on a multitude of experimental parameters, such as the amount of sample analyzed, the temperature ramp rate, the instrument, pan material, and the specific environmental conditions, including atmosphere, means that there is no single cohesive source of ALD-relevant thermochemical data for users of these commercial precursors. We report herein a rigorous non-isothermal TGA study of eight commercial W molecular precursors (Scheme 1) for vapor phase processes highlighting measured TGA temperature windows (*we define this as the region between 5% and 95% of observed mass loss*), enthalpies of sublimation (Δ*H*_sub_), activation energies (*E*_a_), and 1 Torr temperatures. For simplicity and continuity, as most of the precursors are solids at room temperature, we report enthalpies of sublimation (Δ*H*_sub_). However, precursors WH_2_(iPrCp)_2_ and BTBMW are liquids and in these cases, it should be noted we are referring to enthalpies of vaporization (Δ*H*_vap_).

For the remainder of the manuscript, we will use Δ*H*_sub_ as a comprehensive term relating to the enthalpies associated with the precursors entering the gas phase. Additionally, we recognize that commercially available WF_6_ has been successfully used for ALD growth of W and WS_2_ films.^12,13^ Because deposition can produce corrosive HF gas, fluorinated precursors have been omitted from this study.

## 2. Experimental

### 2.1. Materials

Tungsten hexacarbonyl (W(CO)_6_), tetracarbonyl(1,5-cyclooctadiene)tungsten(0) ((1,5-cod)W(CO)_4_), bis(cyclopentadienyl)tungsten(iv) dihydride (WH_2_Cp_2_), and bis(isopropylcyclopentadienyl)tungsten(iv) dihydride (WH_2_(iPrCp)_2_) were obtained from Sigma Aldrich; bis(*t*-butylimido)bis(dimethylamino)tungsten(vi) (BTBMW), and mesitylene tungsten tricarbonyl ((mes)W(CO)_3_) were obtained from Strem Chemicals; WCl_6_ was obtained from Thermo Scientific Chemicals; WCl_5_ was obtained from Entegris. Benzoic acid (Mettler-Toledo calibration substance, analytical standard) was obtained from Sigma-Aldrich, and salicylic acid (certified ACS grade) was obtained from Fisher Chemical.

(mes)W(CO)_3_ was purified by sublimation prior to use based on a published method.^14^ In short, a sublimation apparatus was filled, in a N_2_-atmosphere glovebox, with ∼1 g (mes)W(CO)_3_ and connected to a Schlenk line. The cold finger was connected to a chiller set to 9.5 °C, and the bottom of the sublimation apparatus was submerged into an oil bath which was then heated to 145 °C. Meanwhile, the pressure in the sublimation apparatus was lowered to ∼50 mTorr. After a few hours, the apparatus was taken back into the glovebox and the sublimed yellow product was collected (∼45% of the original loading). All other reagents were used as received without further purification.

### 2.2. Thermogravimetric analysis

All TGA experiments were conducted on an ISI TGA-1000 instrument inside of a N_2_-atmosphere glovebox, with a 5 cm^3^ min^−1^ flow of ultra-high purity N_2_. A platinum pan (surface area 3.44 × 10^−5^ m^2^) was used as a sample holder in all trials. Precursors were spread evenly across the entire surface of the pan. All measurements were conducted non-isothermally at a constant temperature ramp rate of 10 °C min^−1^ and nominal sample loadings of 3, 5, 8, and 10 mg; as well as a constant nominal sample loading of 3 mg with temperature ramp rates of 5, 10, 15, and 20 °C min^−1^. W(CO)_6_ and BTBMW were analyzed from 25 °C up to 200 °C; WH_2_(iPrCp)_2_ was analyzed from 25–300 °C; and (1,5-cod)W(CO)_4_, WH_2_(Cp)_2_, WCl_6_, [WCl_5_]_2_, and (mes)W(CO)_3_ were analyzed from 25–400 °C.

Benzoic acid and salicylic acid standards were analyzed for calibration purposes from RT up to 200 °C, at sample masses of 2 mg, and these trials were repeated three times.

### 2.3. Atomic layer deposition

A Fiji Gen2 PEALD system from Veeco® with an Ebara® multistage dry vacuum pump A30W (pumping speed 3600 l min^−1^) was used to attempt to deposit WO_3_ films on glass slides and silicon wafers with (mes)W(CO)_3_ as the W source. Substrates were ultrasonically cleaned with isopropyl alcohol and deionized water (DIW) and blown dry with nitrogen, which was followed by a 5 min exposure to UV-O_3_ conducted on an Ossila® Model E511. The ultimate base pressure of the ALD chamber was maintained at 5.25 × 10^−7^ Torr with a working pressure of 215 mTorr. The ALD chamber, delivery line and ampoule temperatures are varied according to Table 1.

**Table 1:** Experimental conditions for ALD trials using (mes)W(CO)_3_

Run	Chamber temp. (°C)	Line temp. (°C)	Ampoule temp. (°C)	Pulse time (s)	Oxidant (pulse time, s)
1	175	120	80	0.06	300 W O_2_ plasma (4)
2	175	120	80	0.10	300 W O_2_ plasma (4)
3	175	120	100	0.06	300 W O_2_ plasma (4)
4	175	120	100	0.06	80 sccm O_2_ (10)
5	175	120	100	0.06	H_2_O (0.06)
6	175	150	120	0.06	H_2_O (0.06)
7	175	150	120	0.12	H_2_O (0.06)
a8	175	150	120	0.12	H_2_O (0.06)
a9	175	150	120	0.5	50 W O_2_ plasma (4)
a10	175	150	120	0.5	25 W O_2_ plasma (4)
a11	225	150	120	0.5	25 W O_2_ plasma (4)
a12	225	150	140	0.5	25 W O_2_ plasma (4)

a (8–12) note long form process over 50 cycles using standard alternating pulses of precursor, purge and oxidant, the remaining (1–7) were conducted for ×10 TTT as described in the text.

Previously, one of our groups reported an *in situ* spectroscopic ellipsometry approach for the rapid development of ALD processes. This innovative approach deviates from conventional ALD process optimization recipes in that the pulse time leading to dose saturation is established across a range of temperatures in a single set of experiments. Ten consecutive pulses of precursor are introduced into the chamber with each pulse separated by an argon (Ar) purge. This is then followed by ten consecutive pulses of the oxidant, again with each pulse separated by Ar purge. Thus, in a single sequence of 10 pulses of precursor and oxidant a complete ALD pulse sequence can be mapped. Conducting the same process at different temperatures produces a temperature–(pulse) time–thickness (TTT) graph.^15^

Sets of ALD experiments were conducted with (mes)W(CO)_3_, with de-ionized water (DIW), remote O_2_ plasma, or O_2_ gas as the oxidant. For the present case, ten pulses of (mes)W(CO)_3_ were dosed into the chamber with each pulse separated by a 10 s Ar purge followed by ten consecutive pulses of oxidant each separated by a 10 s Ar purge. Chamber conditions were tested first at 175 °C with ampoule temperatures of 80, 100, and 120 °C as detailed in Table 1 runs 1–7. In the second test the chamber was set to 225 °C with ampoule temperatures of 120 and 140 °C, and growth was attempted with conventional alternating precursor-purge-oxidant-purge pulses (Table 1, runs 8–12). In each test (mes)W(CO)_3_ did build up pressure in the ampule commensurate with volatility but did not yield a reliable ALD growth.

The deposition rate of the film was monitored by *in situ* spectroscopic ellipsometry using a J. A. Woollam® M-2000, with a wavelength range from 273 to 1690 nm. The thickness was modeled using the CompleteEASE® software, consisting of a Cauchy layer optimized with tungsten oxide optical constants on a SiO_2_ substrate layer.

## 3. Results and discussion

**Fig. 2:**
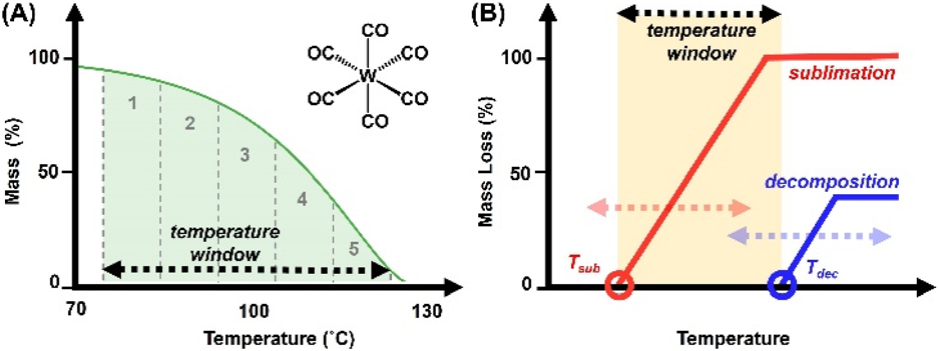
(A) TGA trace of W(CO)_6_ with highlighted “TGA temperature window” and illustration of sequestered zones utilized for calculation of *E*_a_ and Δ*H*_sub_. (B) General schematic to illustrate the correlation of precursor volatility and stability juxtaposed against the potential ALD temperature window.

Precursor behavior was studied by non-isothermal TGA experiments, at a constant temperature ramp rate and with varying sample masses. Results were validated by measuring non-isothermal TGA experiments at fixed (*approximately*) precursor mass, with varying temperature ramp rates. TG curves are plotted as percent mass *versus* temperature. As a figure of comparison, the temperature range in which a precursor undergoes sublimation is designated the “TGA temperature window” of that precursor; specifically, the region between 5% and 95% of observed mass loss (Fig. 2A). Notably, *this is not to be confused with ALD temperature windows as reported in literature*, which are determined by ALD growth processes. The measured TGA temperature window is dependent on the sample mass and the temperature ramp rate. The sublimation kinetics are a function of the surface area of the precursor, which is equal to the surface area of the TGA pan; a platinum pan with a bottom measuring 3.44 × 10^−5^ m^2^. As the surface area remains constant, an increase in the ramp rate shifts the temperature window towards higher temperatures (which are attained more rapidly), while sublimation rates remain limited by the surface area of the TGA pan.

Ultimately, the TGA temperature window serves as a preliminary surrogate for the downstream ALD temperature window. Ideally, precursor volatilization (onset at the temperature of sublimation, *T*_sub_) is complete prior to onset of decomposition (*T*_dec_), *i.e.*, *T*_dec_ sits beyond the TGA temperature window (Fig. 2B). This scenario typically results in little or no residual mass on the TGA pan, and in terms of ALD applicability implies viable delivery of a precursor without thermally induced decomposition which would result in CVD-type growth. The closer the *T*_dec_ lies to the temperature window, the higher the associated residual mass as measured by TGA. The latter can be triggered by high temperature ramp rates and/or higher sample masses, where *T*_dec_ is reached prior to complete volatilization.

**Fig. 3:**
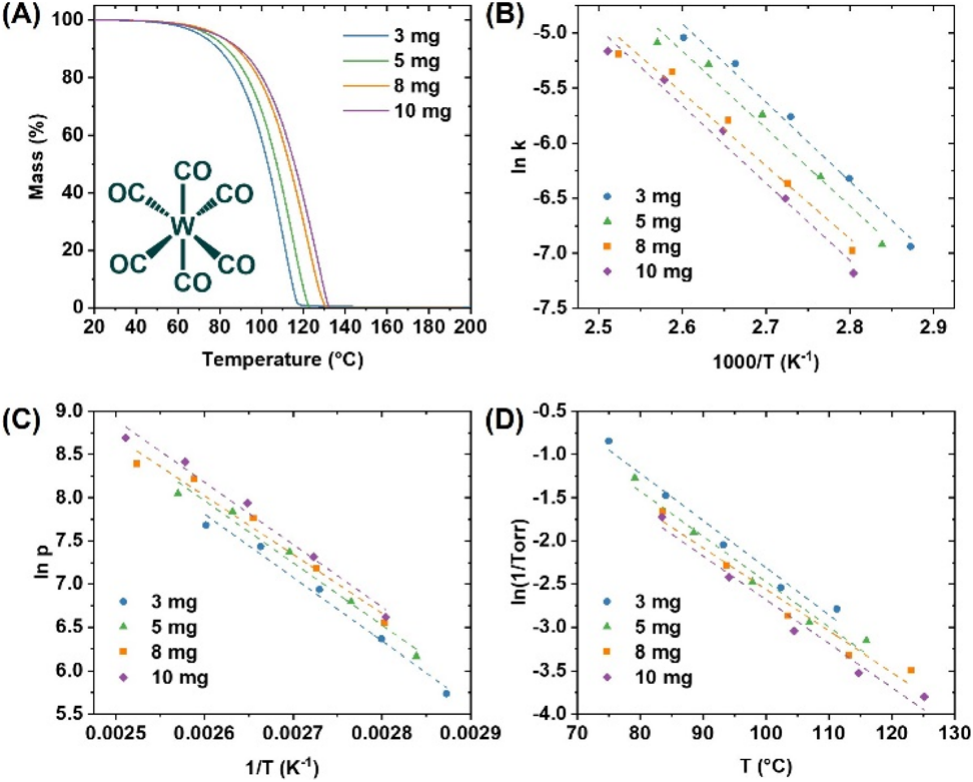
Thermogravimetric analysis of W(CO)_6_: (A) precursor TG trace at a constant temperature ramp rate of 10 °C min^−1^; (B) the Arrhenius plot (*E*_a_ is obtained from the slope of the curves *via* –*E*_a_/*R*); (C) plot of ln *p vs.* 1/*T*, the enthalpy of sublimation is derived from the slope of the straight lines, which is equal to –Δ*H*_sub_/*R*; (D) plot of ln(1/Torr) *vs. T*, used to extrapolate the 1 Torr temperature. *R* = ideal gas constant 8.314 J K^−1^ mol^−1^ (*p* = kPa).

The obtained TGA traces were sectioned off to five equivalent zones within the established TGA temperature window (Fig. 2A), and the extracted values facilitated the calculation of activation energies (*E*_a_) and enthalpies of sublimation (Δ*H*_sub_) for all precursors (Scheme 1). Lower values of *E*_a_ and Δ*H*_sub_ are associated with more volatile precursors. A complete and detailed description of how these values are obtained has been provided in our prior publication.^16^ We note that the measurements performed on the W precursors were conducted in concert with our previously reported Mo precursor chemistry, thus the calibration data for the TGA instrument is identical; as measured with a benzoic acid standard and corroborated with a salicylic acid standard. Finally, a plot of the natural log of 1/Torr *versus T* (°C) was created within the TGA temperature window, to which a linear trendline was fitted and used to calculate the temperature at 1 Torr. An example of these measurements for W(CO)_6_ is highlighted in Fig. 3.

**Fig. 4:**
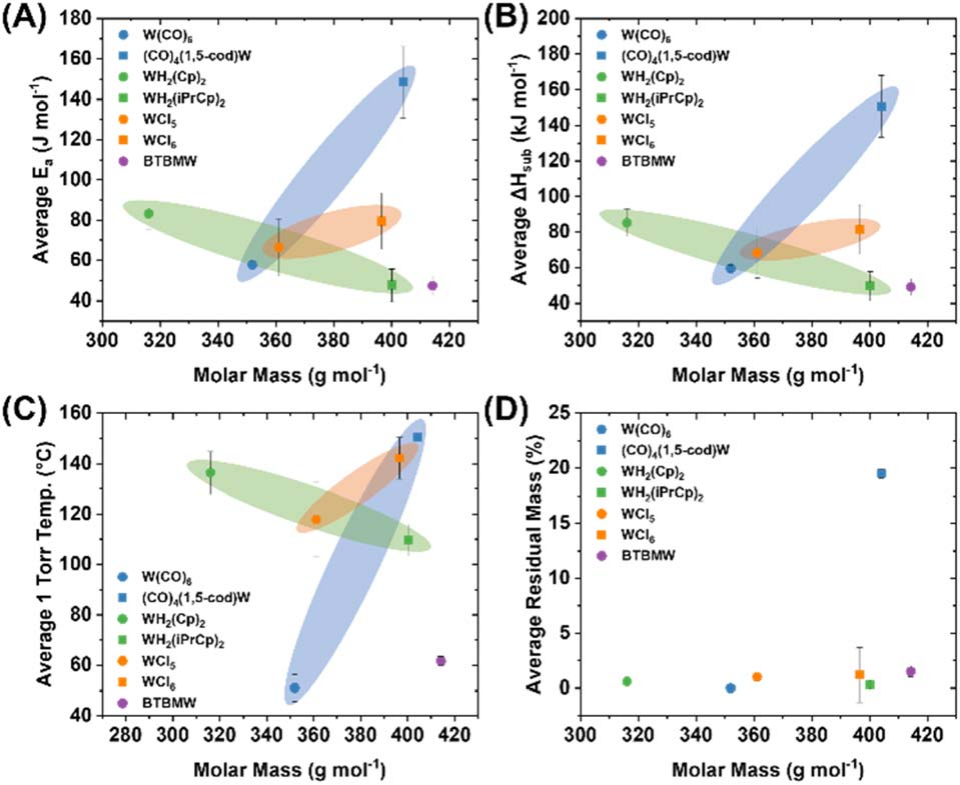
TGA-derived average (A) *E*_a_, (B) Δ*H*_sub_, (C) 1 Torr temperature, and (D) residual mass values from trials varying mass, with fixed 10 °C min^−1^ heating rate, plotted against the molar mass of the precursor molecules.

The average values for *E*_a_ and Δ*H*_sub_ determined for the *ca.* 3, 5, 8, and 10 mg precursor samples are shown in Table 2 (and mapped in Fig. 4) along with their respective derived temperatures at 1 Torr pressure, and the residual masses. In parallel, all molecules were studied by non-isothermal TGA experiments, at a constant mass of *ca.* 3 mg and variable temperature ramp rates of 5, 10, 15, and 20 °C min^−1^. The resulting values appear similar and are included in the ESI† as further validation of the consistency of the methodology used (Table S17†). Direct comparison of values to literature is difficult due to variability introduced by the measurement techniques; nonetheless, the Δ*H*_sub_ values obtained herein are reasonable and correlate to prior reports. For example W(CO)_6_ Δ*H*_sub_ obtained herein is 59.4 ± 1.9 kJ mol^−1^ compared to a broad range from 69.9 ± 4.2 to 78.9 ± 1.1 kJ mol^−1^ from the prior literature.^17–21^ WH_2_Cp_2_ resides even closer to the prior literature with a Δ*H*_sub_ value of 85.3 ± 7.8 kJ mol^−1^, compared to 84.1 ± 1.6 kJ mol^−1^ reported by Dias and coworkers.^22^ Critically, while there may be discrepancies across experimental Δ*H*_sub_ across both the prior literature and the values reported herein, the experimental values across the molecules studied herein are self-consistent which facilitates a direct comparison. This would otherwise be difficult to achieve by collating previously published data across multiple sources.

**Table 2:** TGA-derived *E*_a_, Δ*H*_sub_ and 1 Torr temperature values from trials varying mass, with fixed 10 °C min^−1^ heating rate^a^

Molecule	*E*_a_ (J mol^−1^)	Δ*H*_sub_ (kJ mol^−1^)	*T* at 1 Torr (°C)	Res. mass (%)
W(CO)_6_	57.8 ± 1.9	59.4 ± 1.9	51.0 ± 5.3	0.0 ± 0.0
(1,5-cod)W(CO)_4_	148.5 ± 17.7	150.7 ± 17.3	150.5 ± 1.4	19.5 ± 0.4
WH_2_Cp_2_	83.3 ± 7.8	85.3 ± 7.8	136.4 ± 8.5	0.6 ± 0.0
WH_2_(iPrCp)_2_	47.7 ± 8.0	49.6 ± 8.0	109.8 ± 6.4	0.3 ± 0.3
BTBMW	47.4 ± 4.7	49.0 ± 4.8	61.6 ± 1.8	1.5 ± 0.5
WCl_6_	79.5 ± 14.0	81.3 ± 14.0	142.1 ± 8.3	1.2 ± 2.5
[WCl_5_]_2_	66.5 ± 14.2	68.3 ± 14.2	117.9 ± 14.9	1.0 ± 0.3

a Note (mes)W(CO)_3_ led predominantly to decomposition products with 48.9 ± 0.8% residual mass precluding extraction of sublimation kinetics.

**Fig. 5:**
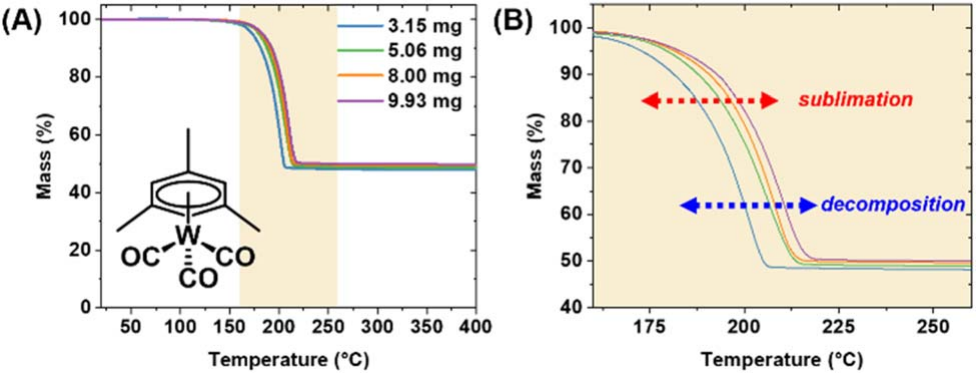
(A) TGA traces of (mes)W(CO)_3_ at varying masses with a temperature ramp rate of 10 °C min^−1^. (B) Close up region where volatility is observed, highlighting overlapping sublimation and decomposition events.

Analysis of most precursors revealed very minimal residual mass. Notably, both (1,5-cod)W(CO)_4_ and (mes)W(CO)_3_ yielded non-trivial amounts of residual mass (19.5 ± 0.4 and 48.9 ± 0.8%, respectively). This is indicative of competing decomposition in addition to sublimation. For (1,5-cod)W(CO)_4_ the residual mass was nonetheless significantly lower than the W metal content of 45.5%, implying a reasonable amount of sublimation prior to decomposition of the ligand framework. This is evident in the well-behaved TGA trace which could be treated similarly as the remaining precursors to extract relevant *E*_a_, Δ*H*_sub_, and 1 Torr temperature values (Fig. S9†). Conversely, the residual mass from (mes)W(CO)_3_ was significantly high at 48.9%, implying a significant amount of decomposition (Fig. 5A). It should be noted that (mes)W(CO)_3_ was purified by sublimation under vacuum,^14^ thus it is well established that it can indeed be volatilized; however, it is clear that the decomposition range overlaps significantly with the TGA temperature window (Fig. 5B). For this reason, reliable *E*_a_, Δ*H*_sub_, and 1 Torr temperature values could not be extracted and were omitted from Table 2 and Fig. 4.

Averaged *E*_a_, Δ*H*_sub_, 1 Torr temperature, and residual mass percentage values were mapped visually as a function of their molar masses (Fig. 4), with precursor molecules categorized according to their general ligand classes; carbonyls [W(CO)_6_, (1,5-cod)W(CO)_4_], cyclopentadienyl [WH_2_Cp_2_, WH_2_(iPrCp)_2_], chlorides [[WCl_5_]_2_, WCl_6_], and amino/imido [BTBMW]. While no clear linear trend emerges among the whole group, there are clear trends within the related molecule groups. Both the carbonyls [W(CO)_6_, (1,5-cod)W(CO)_4_] and chlorides [[WCl_5_]_2_, WCl_6_] show an increase in *E*_a_, Δ*H*_sub_ and 1 Torr temperature commensurate with increasing molar mass. Note, while WCl_5_ is depicted as a dimer [WCl_5_]_2_, that is indicative of the solid state structure, it dissociates to WCl_5_ monomer upon evaporation.^23^ Conversely, among the cyclopentadienyls, WH_2_(iPrCp)_2_ displays significantly lower *E*_a_, Δ*H*_sub_ and 1 Torr temperatures than WH_2_Cp_2_ despite a molar mass difference of +84.16 g mol^−1^. Namely, the *E*_a_ is 43% lower, the Δ*H*_sub_ is 42% lower, while the molar mass is 27% higher for WH_2_(iPrCp)_2_. The presence of the iPr group comparatively lowers the symmetry of the molecule rendering it liquid at room temperature, compared to WH_2_Cp_2_ which is a crystalline solid at room temperature, with a reported melting point at ∼115 °C.^24^ BTBMW, another liquid at room temperature (likely aided by the presence of the *t*Bu groups), displayed the lowest *E*_a_, Δ*H*_sub_ and 1 Torr temperatures despite having the highest molar mass of all molecules tested (414.23 g mol^−1^). It is noteworthy how much of an impact the introduction of bulky functional groups that can inhibit crystalline packing, and in this case render the material liquid, has on achieving rapid volatility, as compared to simply lowering the molecular mass. The latter is typically done by choosing smaller ligands, which are often more symmetrical and result in crystalline solids at room temperature.

Among the non-halides, and to the best of our knowledge, W(CO)_6_,^25–28^ WH_2_Cp_2_,^25,29^ WH_2_(iPrCp)_2_,^30,31^ and BTBMW^25,32–39^ have been successfully utilized for ALD processes, whereas all have been utilized for CVD^39–48^ (halides included^48–54^). While (1,5-cod)W(CO)_4_ exhibits higher-than-average *E*_a_, Δ*H*_sub_ and residual mass values, the temperature window falls within currently useable range, and is likely adaptable to an ALD growth process. The higher-than-average *E*_a_ and Δ*H*_sub_ values can be readily mitigated through the use of low vapor pressure ALD precursor delivery systems. On the contrary, and as noted above, the residual mass from (mes)W(CO)_3_ was significantly high which implied a significant amount of decomposition within the sublimation temperature window (Fig. 5A). Based on the implication of TGA screening as a prognosticator for ALD precursor viability, this result implies that (mes)W(CO)_3_ would not be a feasible precursor. However, as the molecule was readily sublimed under vacuum (using standard Schlenk line techniques with a standard vacuum set up at ∼50 mTorr) for purification, it warranted further testing for validation.

To test viability for ALD growth of WO_*x*_, experiments were conducted with (mes)W(CO)_3_, and DIW, remote O_2_ plasma, and O_2_ gas as the oxidant. Further details are found in the experimental section. Chamber temperatures were tested initially at 175 °C with ampoule/precursor temperatures of 80, 100, and 120 °C. Subsequently the chamber was set to 225 °C and the ampoule temperatures at 120 and 140 °C. In each test there was a noted buildup of pressure in the ampule commensurate with generation of volatile compounds, but ultimately reliable ALD growth could not be achieved under any of the conditions noted. Based on the ALD results, and the TGA trials, it appears that precursor decomposition is the dominant route within the observed window of volatility (Fig. 5B), and very likely, the majority of pressure build up within the ampoule was the generation of volatile organics and CO. Thus while (mes)W(CO)_3_ can be purified at elevated temperature by sublimation under continuous vacuum, TGA analysis under N_2_ flow (instead of reduced pressure), and incubation within an ALD ampoule commensurate with pulsed delivery leads in both latter cases predominantly to decomposition products. Thus, the TGA analysis herein is adequately predictive for the non-feasibility of (mes)W(CO)_3_ for standard ALD but does not preclude potential applications in various CVD processes where the precursor can be delivered continuously or using a stabilizing solvent carrier.

## 4. Conclusions

The volatility and thermal stability of eight commercially available CVD and ALD molecular precursors based on W were evaluated by non-isothermal TGA experiments. Precursor ceramic yields and ALD temperature windows were obtained directly from the TGA plots. Activation energy (*E*_a_), enthalpy of sublimation (Δ*H*_sub_), and 1 Torr temperature values were determined from the TGA data. Because these values are dependent on experimental set-up, a direct comparison of such values collected across variable literature reports is complicated. Our report provides a unified, and rigorous comparison of such values, which can serve as a quick reference map of temperature windows to rapidly match to desired temperature regimes for W-based film growth. A closer look at volatility trends among the various molecule ligand groupings, and at large, reveals the importance of ligand design choice, namely introduction of bulky, symmetry-lowering substituents to prevent crystallinity and afford low Δ*H*_sub_ values. Moreover, the predicting factor of TGA screening was juxtaposed with known material volatility when the TGA trials for (mes)W(CO)_3_ pointed to low feasibility for ALD application. Therein, attempted ALD growth of WO_*x*_ under a variety of conditions yielded no thin films, validating the TGA screening approach.

## Data availability

All measured TGA experiments are provided as plots in the ESI.† Data generated by TGA measurements^55^ is available from the Materials Data Facility.^56,57^

## Conflicts of interest

There are no conflicts to declare.

## Supplementary Material

RA-014-D4RA07284G-s001
