# Supplementary material for: Thermogravimetric analysis of commercial tungsten molecular precursors for vapor phase deposition processes
Source: RSC Adv. 2024 Dec 19;14(54):39867–73. doi: 10.1039/d4ra07284g (PMC11656762; doi:10.1039/d4ra07284g)
Supplement: RA-014-D4RA07284G-s001 [file RA-014-D4RA07284G-s001.pdf]

Supplementary Information (SI) for RSC Advances.
This journal is © The Royal Society of Chemistry 2024

# Thermogravimetric analysis of commercial tungsten molecular precursors for vapor phase deposition processes

Taylor M. Currie,[a,b] Terrick McNealy-James,[b,c] S. Novia Berriel,[b,c] Konstantin Preradovic,[a] Alfred P. Sattelberger,[a] Parag Banerjee,[b,c,d,e] and Titel Jurca,*[a,b,d]

[a.] *Department of Chemistry, University of Central Florida, Orlando, Florida 32816, USA*
[b.] *Renewable Energy and Chemical Transformations Cluster, University of Central Florida, Orlando, Florida 32816, USA*
[c.] *Department of Materials Science and Engineering, University of Central Florida, Orlando, Florida 32816, USA*
[d.] *NanoScience & Technology Center, University of Central Florida, Orlando, Florida 32826, USA*
[e.] *Florida Solar Energy Center, University of Central Florida, Cocoa, Florida 32922, USA*

## Table of contents

1. Thermogravimetric analysis of $W(CO)_6$........ S2-S4
2. Thermogravimetric analysis of BTBMW........ S5-S7
3. Thermogravimetric analysis of (1,5-cod)$W(CO)_4$........ S8-S10
4. Thermogravimetric analysis of $WH_2(iPrCp)_2$........ S11-S13
5. Thermogravimetric analysis of $WH_2(Cp)_2$........ S14-S16
6. Thermogravimetric analysis of $[WCl_5]_2$........ S17-S19
7. Thermogravimetric analysis of $WCl_6$........ S20-S22
8. Thermogravimetric analysis of (mes)$W(CO)_3$........ S23
9. Comparison of TGA-derived values for all precursors........ S24

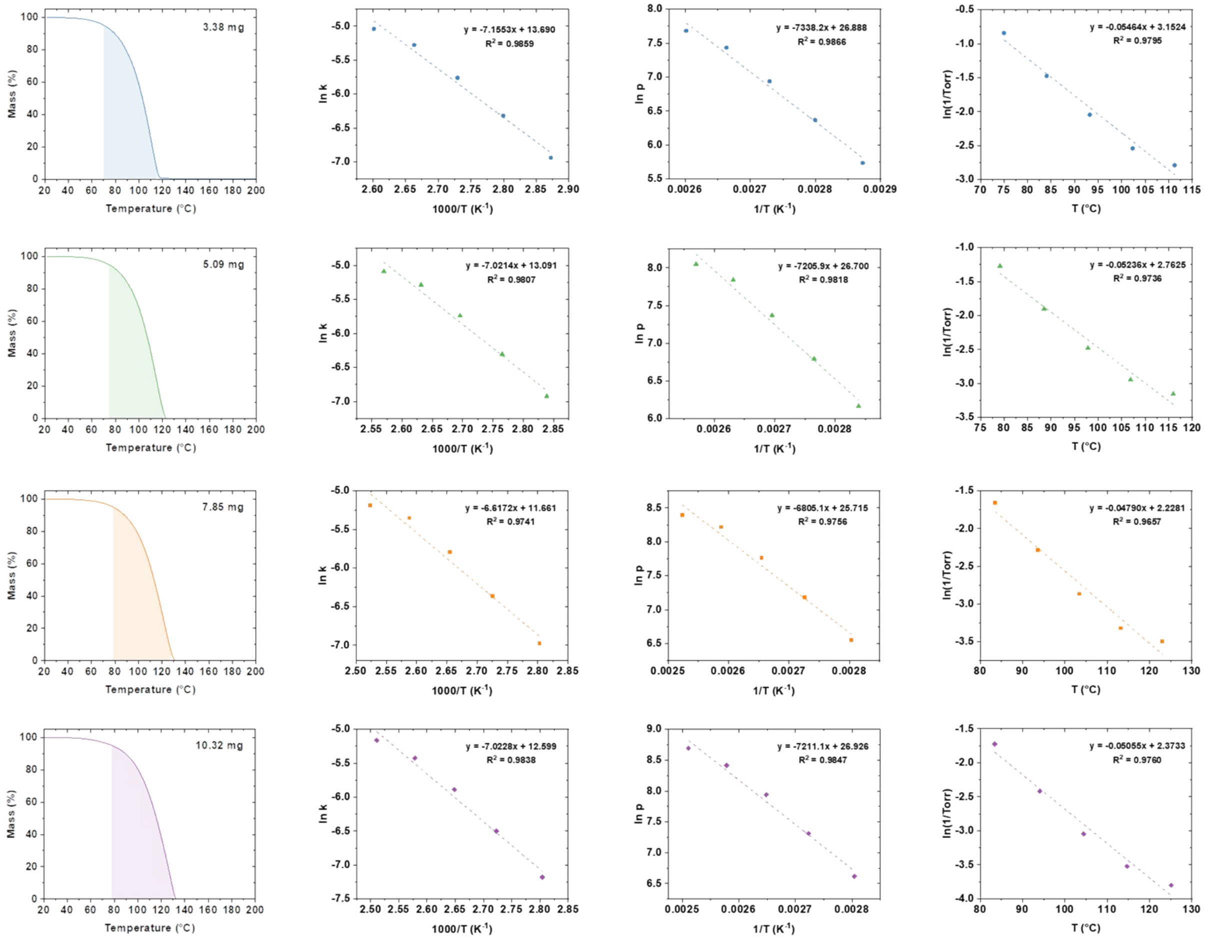


**Figure S1.** Thermogravimetric analysis of $W(CO)_6$. TG traces at constant temperature ramp rate of 10 °C/min and nominally 3 (blue), 5 (green), 8 (orange), and 10 (purple) mg (with actual masses displayed on graphs). Shaded regions underneath TG traces represent TGA temperature windows. Plots of ln *k* vs 1000/*T*, ln *p* vs 1/*T*, and ln(1/Torr) vs *T* used to derive $E_a$, $\Delta H_{sub}$, and *T* at 1 Torr, respectively. Equation of line of best fit and $R^2$ value displayed on each of these plots.

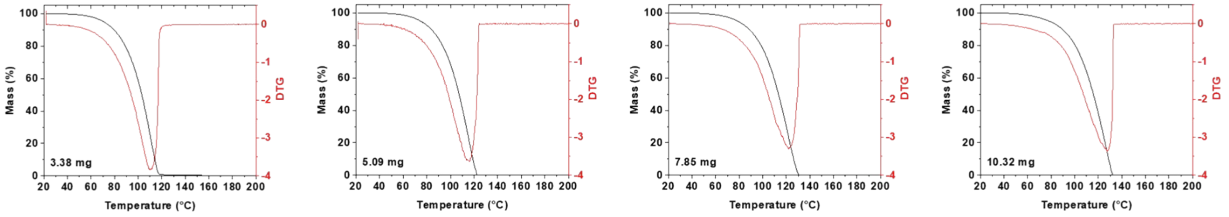


**Figure S2.** Thermogravimetric analysis of $W(CO)_6$. TG traces at constant temperature ramp rate of 10 °C/min and variable masses with derivative thermogravimetric (DTG) traces shown.

**Table S1.** Measured and calculated thermophysical parameters from TGA of $W(CO)_6$ at constant temperature ramp rate of 10 °C/min and variable masses.

| Sample mass (mg) | 3.38 | 5.09 | 7.85 | 10.32 |
|---|---|---|---|---|
| Slope (Arrhenius plot) | -7.16 | -7.02 | -6.62 | -7.02 |
| $R^2$ (Arrhenius plot) | 0.99 | 0.98 | 0.97 | 0.98 |
| $E_a$ (J/mol) | 59.49 | 58.38 | 55.02 | 58.39 |
| $R^2$ (Clausius-Clapeyron plot) | 0.99 | 0.98 | 0.98 | 0.98 |
| $\Delta H_{sub}$ (kJ/mol) | 61.01 | 59.91 | 56.58 | 59.96 |
| $T$ at 1 Torr (°C) | 57.69 | 52.76 | 46.52 | 46.95 |
| $T$ window (°C) | 71-116 | 75-120 | 79-128 | 78-130 |
| Residual mass (%) | 0.00 | 0.00 | 0.00 | 0.00 |

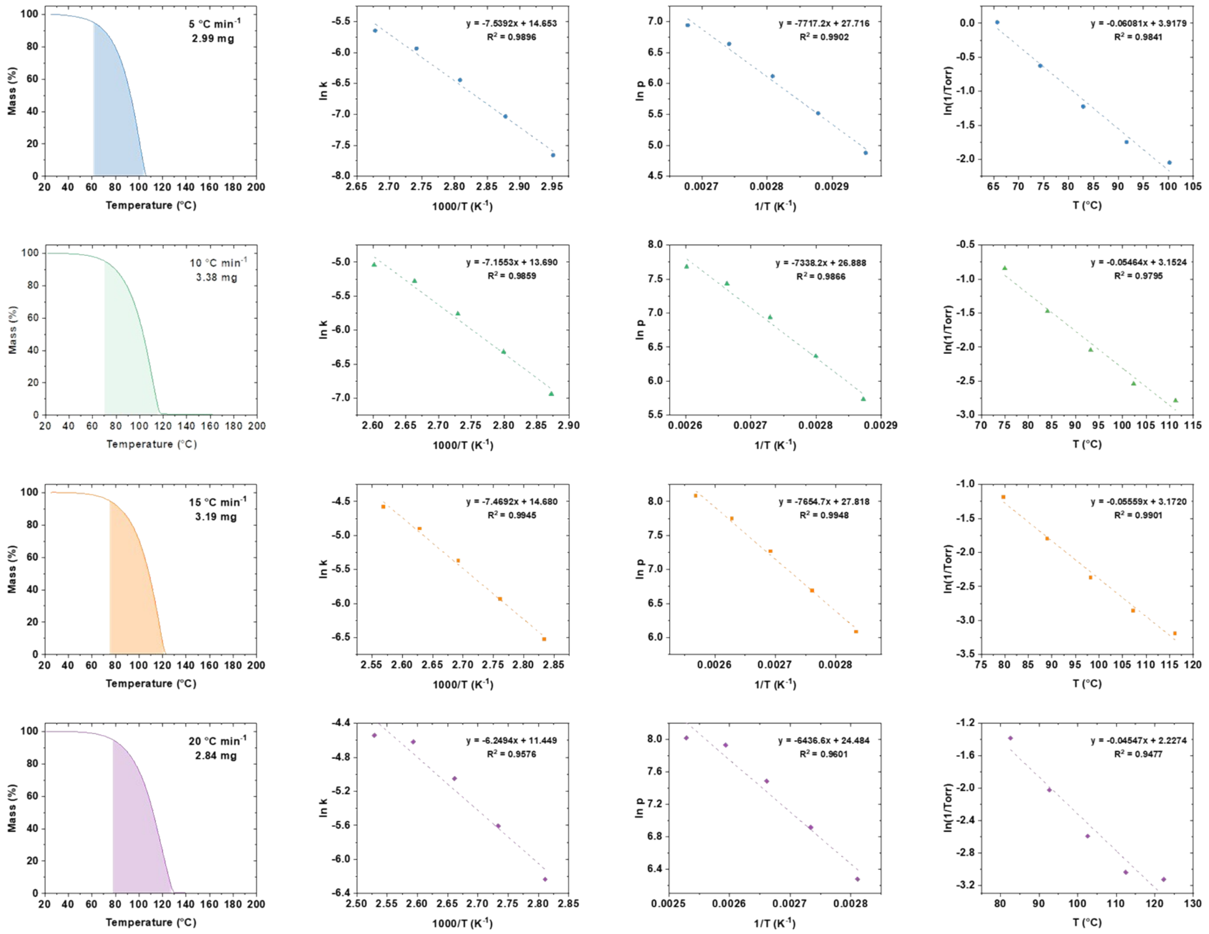


**Figure S3.** Thermogravimetric analysis of $W(CO)_6$. TG traces at nominally 3 mg and variable temperature ramp rates of 5 (blue), 10 (green), 15 (orange), and 20 (purple) °C/min (with actual masses displayed on graphs). Shaded regions underneath TG traces represent TGA temperature windows. Plots of ln $k$ vs 1000/$T$, ln $p$ vs 1/$T$, and ln(1/Torr) vs $T$ used to derive $E_a$, $\Delta H_{sub}$, and $T$ at 1 Torr, respectively. Equation of line of best fit and $R^2$ value displayed on each of these plots.

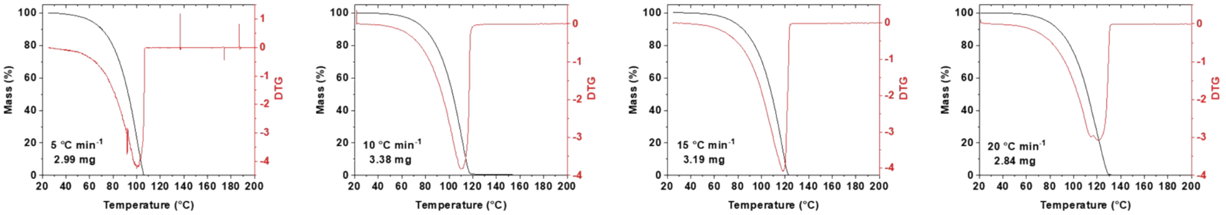


**Figure S4.** Thermogravimetric analysis of $W(CO)_6$. TG traces at nominally 3 mg and variable temperature ramp rates with derivative thermogravimetric (DTG) traces shown.

**Table S2.** Measured and calculated thermophysical parameters from TGA of $W(CO)_6$ at nominally 3 mg and variable temperature ramp rates.

| Heating rate (°C/min) | 5 | 10 | 15 | 20 |
|---|---|---|---|---|
| Sample mass (mg) | 2.99 | 3.38 | 3.19 | 2.84 |
| Slope (Arrhenius plot) | -7.54 | -7.16 | -7.47 | -6.25 |
| $R^2$ (Arrhenius plot) | 0.99 | 0.99 | 0.99 | 0.96 |
| $E_a$ (J/mol) | 62.68 | 59.49 | 62.10 | 51.96 |
| $R^2$ (Clausius-Clapeyron plot) | 0.99 | 0.99 | 0.99 | 0.96 |
| $\Delta H_{sub}$ (kJ/mol) | 64.16 | 61.01 | 63.65 | 53.52 |
| $T$ at 1 Torr (°C) | 64.43 | 57.69 | 57.06 | 48.99 |
| $T$ window (°C) | 64-104 | 71-116 | 75-121 | 78-127 |
| Residual mass (%) | 0.00 | 0.00 | 0.00 | 0.08 |

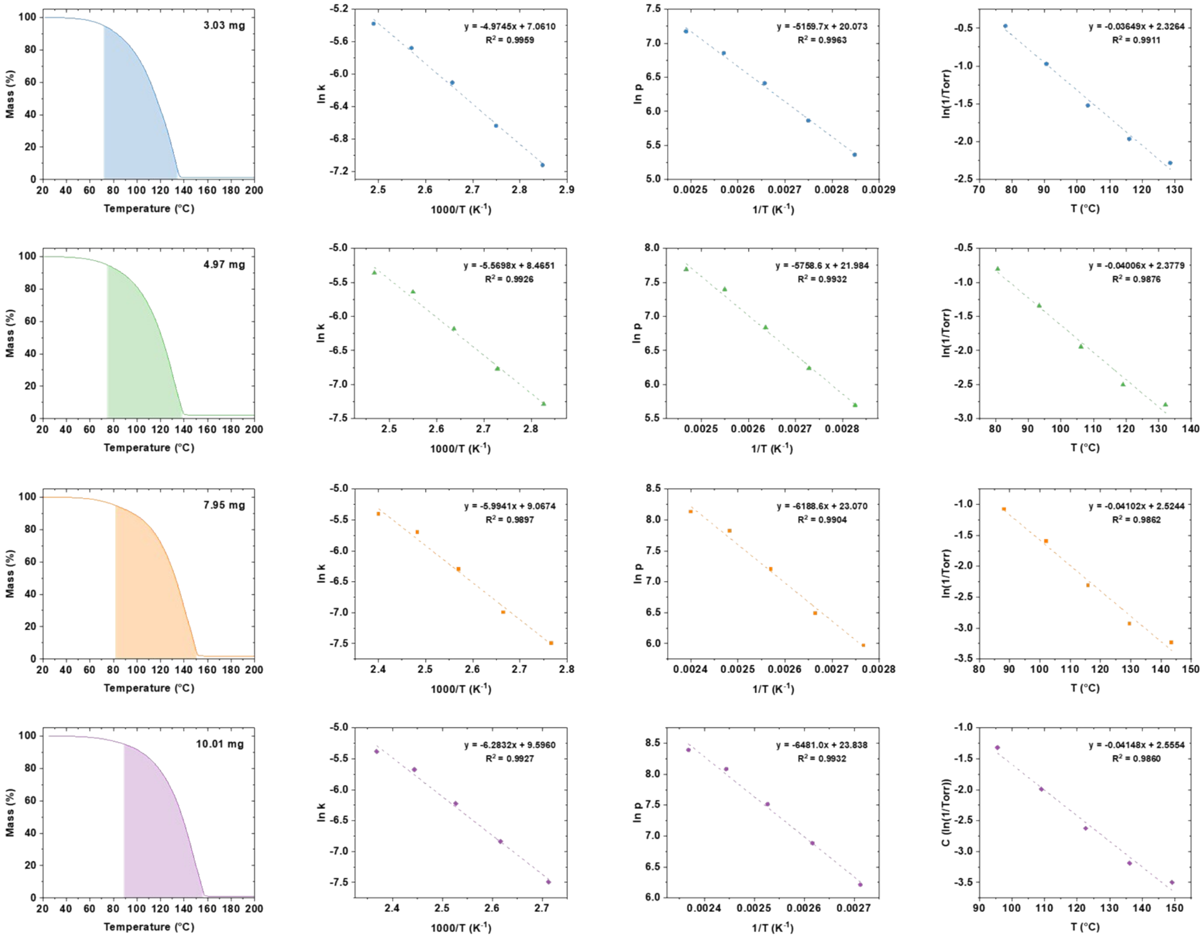


**Figure S5.** Thermogravimetric analysis of BTBMW. TG traces at constant temperature ramp rate of 10 °C/min and nominally 3 (blue), 5 (green), 8 (orange), and 10 (purple) mg (with actual masses displayed on graphs). Shaded regions underneath TG traces represent TGA temperature windows. Plots of ln *k* vs 1000/*T*, ln *p* vs 1/*T*, and ln(1/Torr) vs *T* used to derive $E_a$, $\Delta H_{sub}$, and *T* at 1 Torr, respectively. Equation of line of best fit and $R^2$ value displayed on each of these plots.

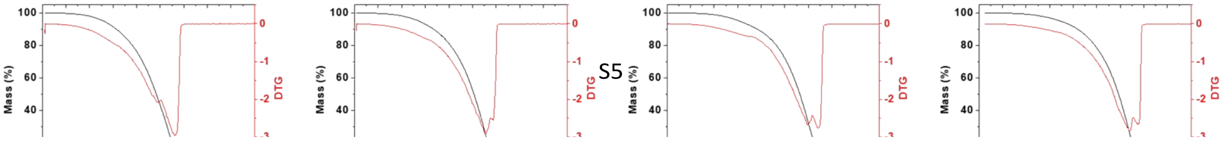


**Figure S6.** Thermogravimetric analysis of BTBMW. TG traces at constant temperature ramp rate of 10 °C/min and variable masses with derivative thermogravimetric (DTG) traces shown.

**Table S3.** Measured and calculated thermophysical parameters from TGA of BTBMW at constant temperature ramp rate of 10 °C/min and variable masses.

| Sample mass (mg) | 3.03 | 4.97 | 7.95 | 10.01 |
|---|---|---|---|---|
| Slope (Arrhenius plot) | -4.97 | -5.57 | -5.99 | -6.28 |
| $R^2$ (Arrhenius plot) | 1.00 | 0.99 | 0.99 | 0.99 |
| $E_a$ (J/mol) | 41.36 | 46.31 | 49.83 | 52.24 |
| $R^2$ (Clausius-Clapeyron plot) | 1.00 | 0.99 | 0.99 | 0.99 |
| $\Delta H_{sub}$ (kJ/mol) | 42.90 | 47.88 | 51.46 | 53.89 |
| $T$ at 1 Torr (°C) | 63.75 | 59.36 | 61.54 | 61.61 |
| $T$ window (°C) | 72-135 | 74-139 | 81-150 | 89-156 |
| Residual mass (%) | 1.23 | 2.09 | 1.64 | 0.92 |

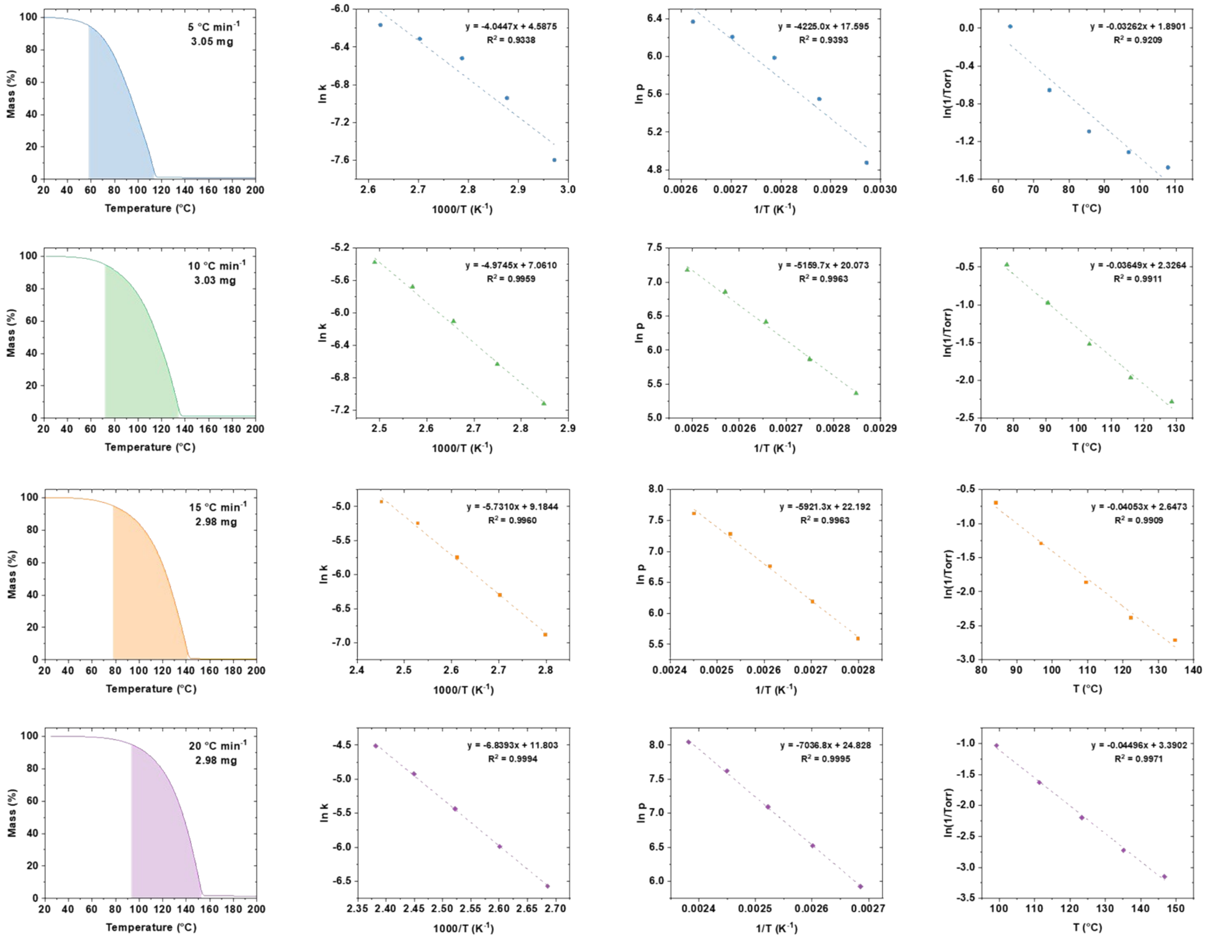


**Figure S7.** Thermogravimetric analysis of BTBMW. TG traces at nominally 3 mg and variable temperature ramp rates of 5 (blue), 10 (green), 15 (orange), and 20 (purple) °C/min (with actual masses displayed on graphs). Shaded regions underneath TG traces represent TGA temperature windows. Plots of ln $k$ vs 1000/$T$, ln $p$ vs 1/$T$, and ln(1/Torr) vs $T$ used to derive $E_a$, $\Delta H_{sub}$, and $T$ at 1 Torr, respectively. Equation of line of best fit and $R^2$ value displayed on each of these plots.

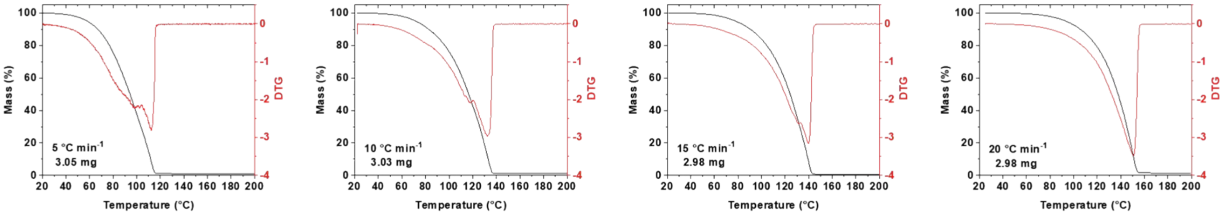


**Figure S8.** Thermogravimetric analysis of BTBMW. TG traces at nominally 3 mg and variable temperature ramp rates with derivative thermogravimetric (DTG) traces shown.

**Table S4.** Measured and calculated thermophysical parameters from TGA of BTBMW at nominally 3 mg and variable temperature ramp rates.

| Heating rate (°C /min) | 5 | 10 | 15 | 20 |
|---|---|---|---|---|
| Sample mass (mg) | 3.05 | 3.03 | 2.98 | 2.98 |
| Slope (Arrhenius plot) | -4.04 | -4.97 | -5.73 | -6.84 |
| $R^2$ (Arrhenius plot) | 0.93 | 1.00 | 1.00 | 1.00 |
| $E_a$ (J/mol) | 33.63 | 41.36 | 47.65 | 56.86 |
| $R^2$ (Clausius-Clapeyron plot) | 0.94 | 1.00 | 1.00 | 1.00 |
| $\Delta H_{sub}$ (kJ/mol) | 35.13 | 42.90 | 49.23 | 58.51 |
| $T$ at 1 Torr (°C) | 57.94 | 63.75 | 65.32 | 74.27 |
| $T$ window (°C) | 58-114 | 72-135 | 78-141 | 93-153 |
| Residual mass (%) | 0.86 | 1.23 | 0.58 | 1.49 |

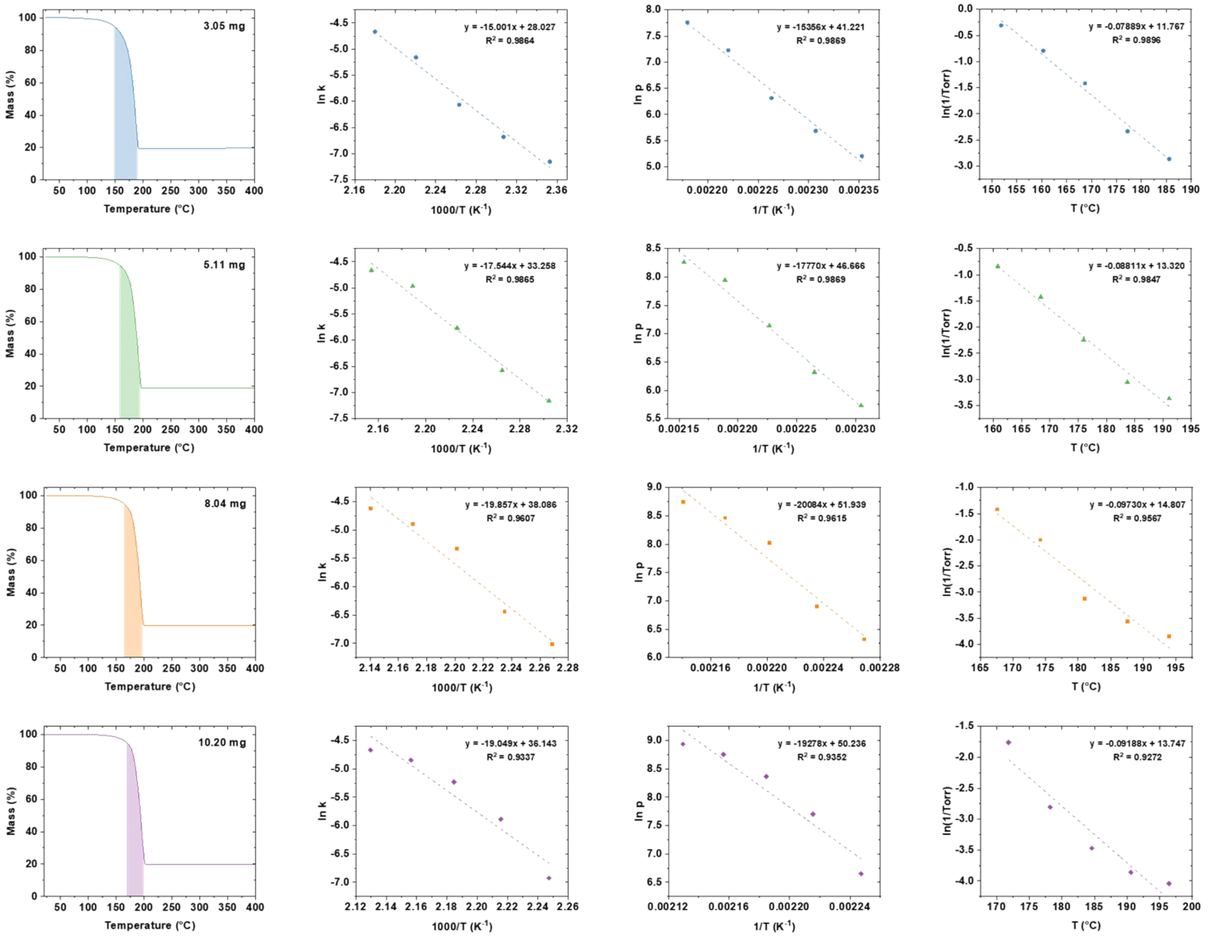


**Figure S9.** Thermogravimetric analysis of (1,5-cod)W$(CO)_4$. TG traces at constant temperature ramp rate of 10 °C/min and nominally 3 (blue), 5 (green), 8 (orange), and 10 (purple) mg (with actual masses displayed on graphs). Shaded regions underneath TG traces represent TGA temperature windows. Plots of ln *k* vs 1000/*T*, ln *p* vs 1/*T*, and ln(1/Torr) vs *T* used to derive $E_a$, $\Delta H_{sub}$, and *T* at 1 Torr, respectively. Equation of line of best fit and $R^2$ value displayed on each of these plots.

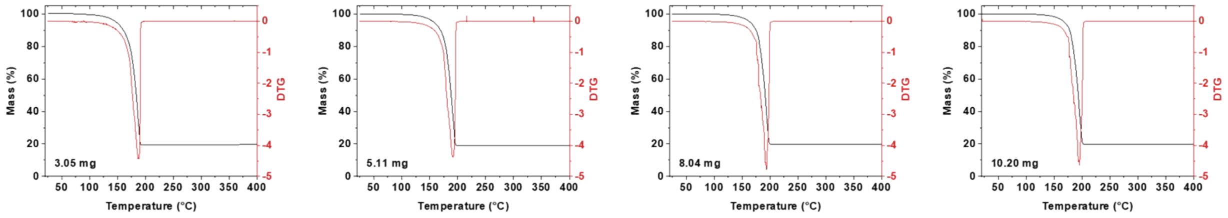


**Figure S10.** Thermogravimetric analysis of (1,5-cod)W$(CO)_4$. TG traces at constant temperature ramp rate of 10 °C/min and variable masses with derivative thermogravimetric (DTG) traces shown.

**Table S5.** Measured and calculated thermophysical parameters from TGA of (1,5-cod)W$(CO)_4$ at constant temperature ramp rate of 10 °C/min and variable masses.

| Sample mass (mg) | 3.05 | 5.11 | 8.04 | 10.20 |
|---|---|---|---|---|
| Slope (Arrhenius plot) | -15.00 | -17.54 | -19.86 | -19.05 |
| $R^2$ (Arrhenius plot) | 0.99 | 0.99 | 0.96 | 0.93 |
| $E_a$ (J/mol) | 124.72 | 145.86 | 165.09 | 158.37 |
| $R^2$ (Clausius-Clapeyron plot) | 0.99 | 0.99 | 0.96 | 0.94 |
| $\Delta H_{sub}$ (kJ/mol) | 127.68 | 147.75 | 166.99 | 160.29 |
| $T$ at 1 Torr (°C) | 149.16 | 151.17 | 152.18 | 149.62 |
| $T$ window (°C) | 148-190 | 157-195 | 164-197 | 169-199 |
| Residual mass (%) | 19.67 | 18.97 | 19.65 | 19.75 |

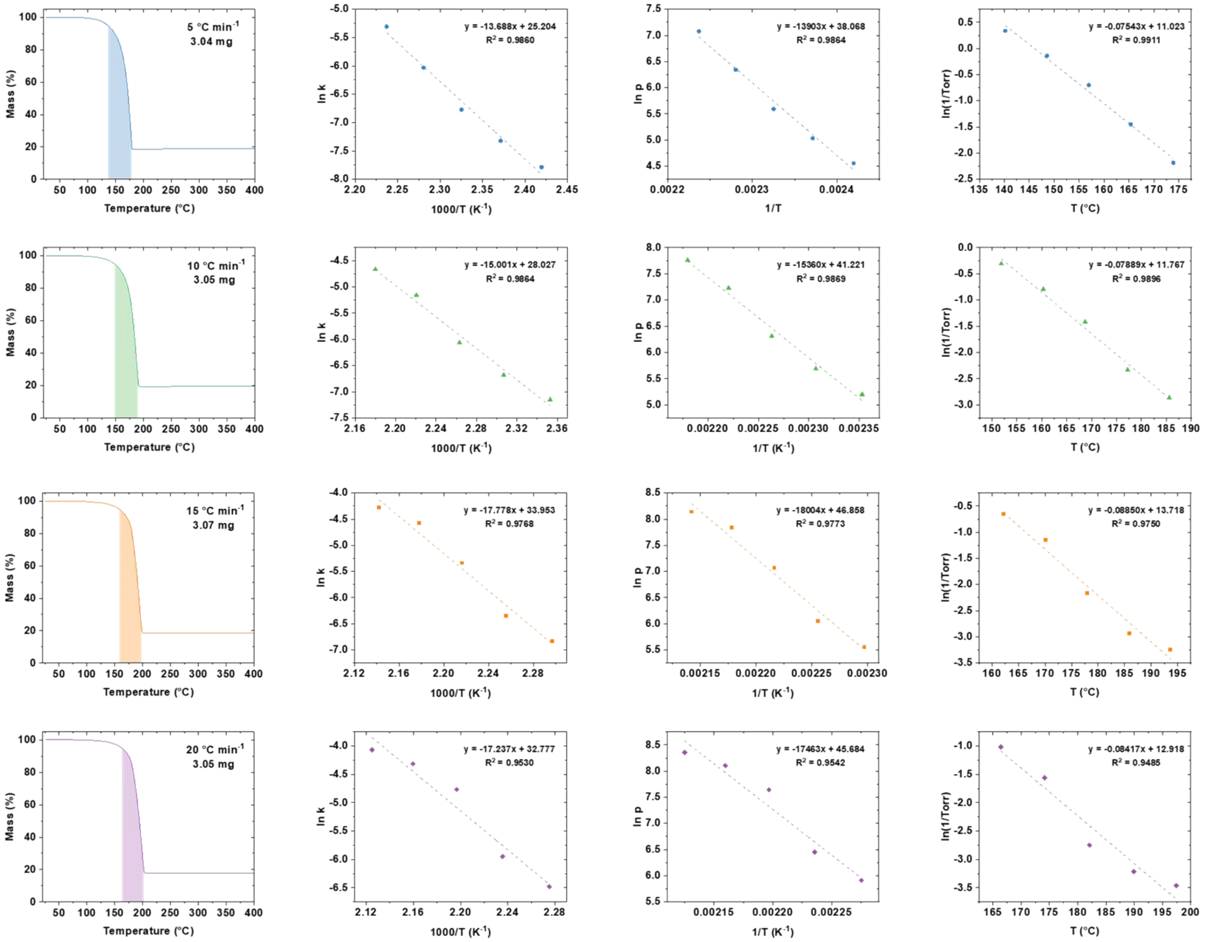


**Figure S11.** Thermogravimetric analysis of (1,5-cod)W$(CO)_4$. TG traces at nominally 3 mg and variable temperature ramp rates of 5 (blue), 10 (green), 15 (orange), and 20 (purple) °C/min (with actual masses displayed on graphs). Shaded regions underneath TG traces represent TGA temperature windows. Plots of ln $k$ vs 1000/$T$, ln $p$ vs 1/$T$, and ln(1/Torr) vs $T$ used to derive $E_a$, $\Delta H_{sub}$, and $T$ at 1 Torr, respectively. Equation of line of best fit and $R^2$ value displayed on each of these plots.

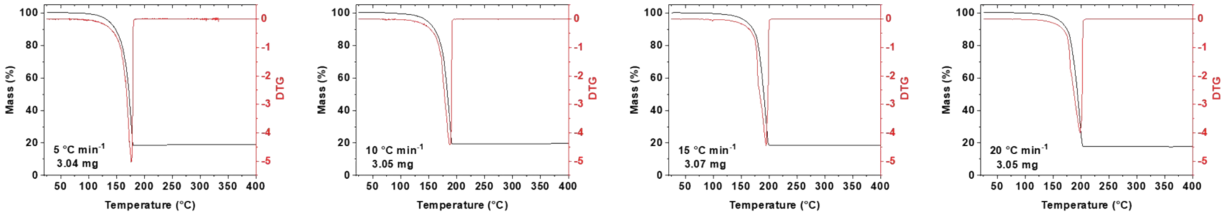


**Figure S12.** Thermogravimetric analysis of (1,5-cod)W$(CO)_4$. TG TG traces at nominally 3 mg and variable temperature ramp rates with derivative thermogravimetric (DTG) traces shown.

**Table S6.** Measured and calculated thermophysical parameters from TGA of (1,5-cod)W$(CO)_4$ at nominally 3 mg and variable temperature ramp rates.

| Heating rate (°C/min) | 5 | 10 | 15 | 20 |
|---|---|---|---|---|
| Sample mass (mg) | 3.04 | 3.05 | 3.07 | 3.05 |
| Slope (Arrhenius plot) | -13.69 | -15.00 | -17.78 | -17.24 |
| $R^2$ (Arrhenius plot) | 0.99 | 0.99 | 0.98 | 0.95 |
| $E_a$ (J/mol) | 113.80 | 124.72 | 147.81 | 143.31 |
| $R^2$ (Clausius-Clapeyron plot) | 0.99 | 0.99 | 0.98 | 0.95 |
| $\Delta H_{sub}$ (kJ/mol) | 115.60 | 127.71 | 149.69 | 145.20 |
| $T$ at 1 Torr (°C) | 146.14 | 149.16 | 155.01 | 153.48 |
| $T$ window (°C) | 136-178 | 148-190 | 158-197 | 163-201 |
| Residual mass (%) | 19.10 | 19.67 | 18.68 | 17.65 |

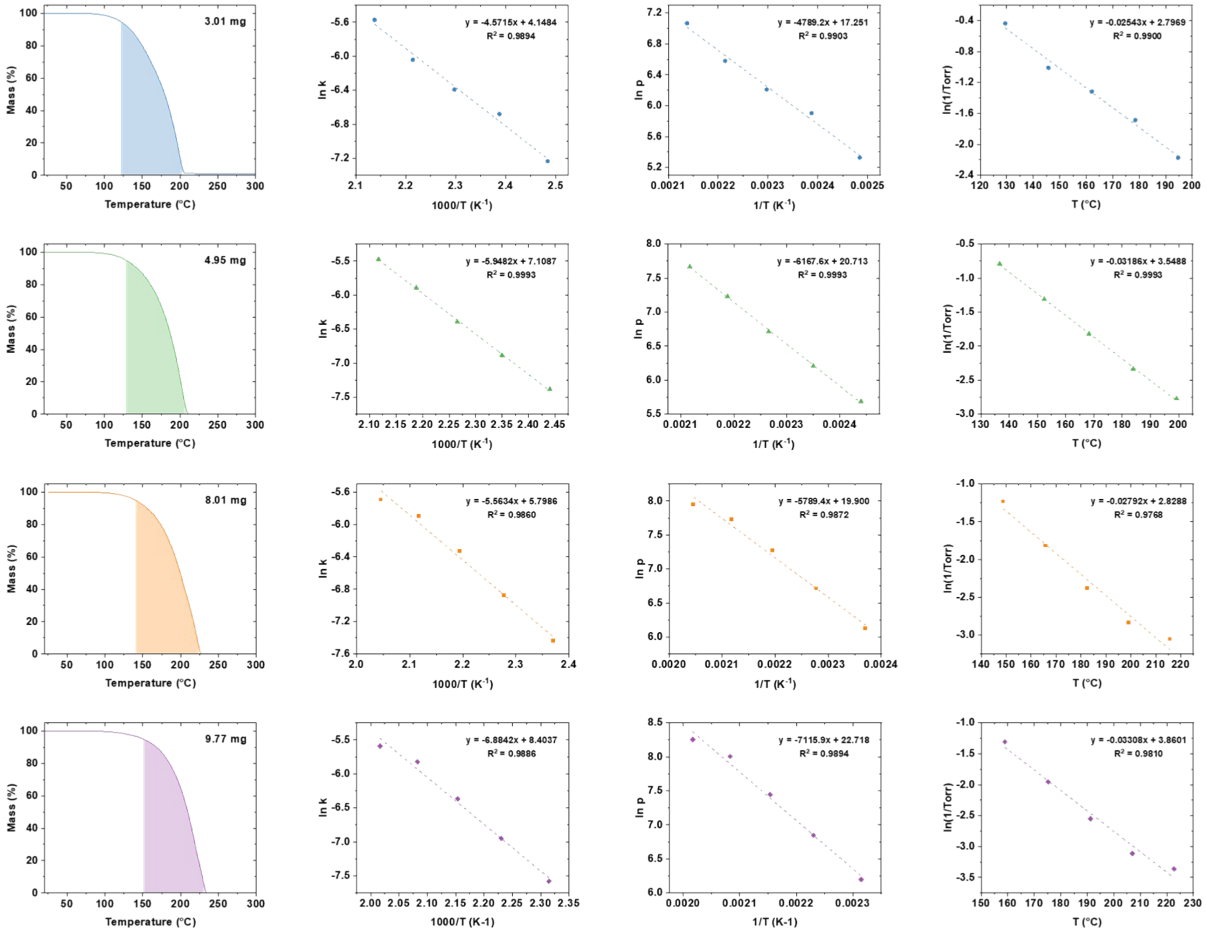


**Figure S13.** Thermogravimetric analysis of $WH_2(iPrCp)_2$. TG traces at constant temperature ramp rate of 10 °C/min and nominally 3 (blue), 5 (green), 8 (orange), and 10 (purple) mg (with actual masses displayed on graphs). Shaded regions underneath TG traces represent TGA temperature windows. Plots of ln *k* vs 1000/*T*, ln *p* vs 1/*T*, and ln(1/Torr) vs *T* used to derive $E_a$, $\Delta H_{sub}$, and *T* at 1 Torr, respectively. Equation of line of best fit and $R^2$ value displayed on each of these plots.

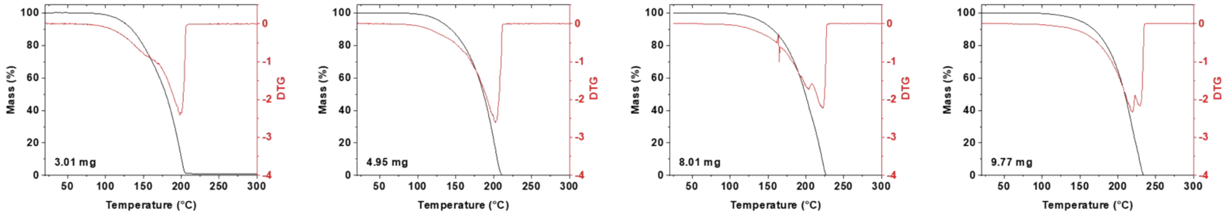


**Figure S14.** Thermogravimetric analysis of $WH_2(iPrCp)_2$. Thermogravimetric analysis of $W(CO)_6$. TG traces at constant temperature ramp rate of 10 °C/min and variable masses with derivative thermogravimetric (DTG) traces shown.

**Table S7.** Measured and calculated thermophysical parameters from TGA of $WH_2(iPrCp)_2$ at constant temperature ramp rate of 10 °C/min and variable masses.

| Sample mass (mg) | 3.01 | 4.95 | 8.01 | 9.77 |
|---|---|---|---|---|
| Slope (Arrhenius plot) | -4.57 | -5.95 | -5.56 | -6.88 |
| $R^2$ (Arrhenius plot) | 0.99 | 1.00 | 0.99 | 0.99 |
| $E_a$ (J/mol) | 38.01 | 49.45 | 46.25 | 57.24 |
| $R^2$ (Clausius-Clapeyron plot) | 0.99 | 1.00 | 0.99 | 0.99 |
| $\Delta H_{sub}$ (kJ/mol) | 39.82 | 51.28 | 48.14 | 59.17 |
| $T$ at 1 Torr (°C) | 109.98 | 111.39 | 101.32 | 116.69 |
| $T$ window (°C) | 121-203 | 129-207 | 140-224 | 151-231 |
| Residual mass (%) | 0.80 | 0.13 | 0.30 | 0.08 |

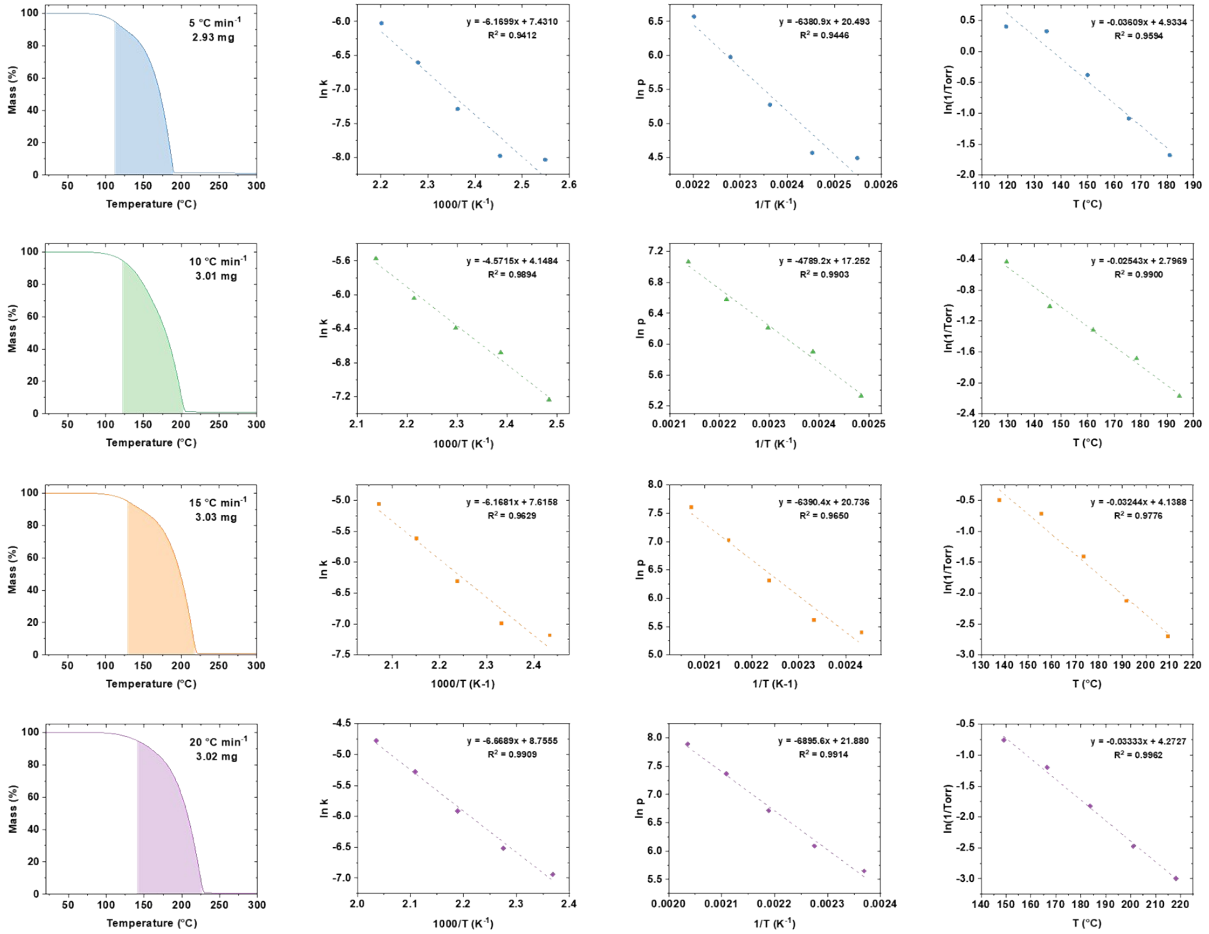


**Figure S15.** Thermogravimetric analysis of $WH_2(iPrCp)_2$. TG traces at nominally 3 mg and variable temperature ramp rates of 5 (blue), 10 (green), 15 (orange), and 20 (purple) °C/min (with actual masses displayed on graphs). Shaded regions underneath TG traces represent TGA temperature windows. Plots of ln *k* vs 1000/*T*, ln *p* vs 1/*T*, and ln(1/Torr) vs *T* used to derive $E_a$, $\Delta H_{sub}$, and *T* at 1 Torr, respectively. Equation of line of best fit and $R^2$ value displayed on each of these plots.

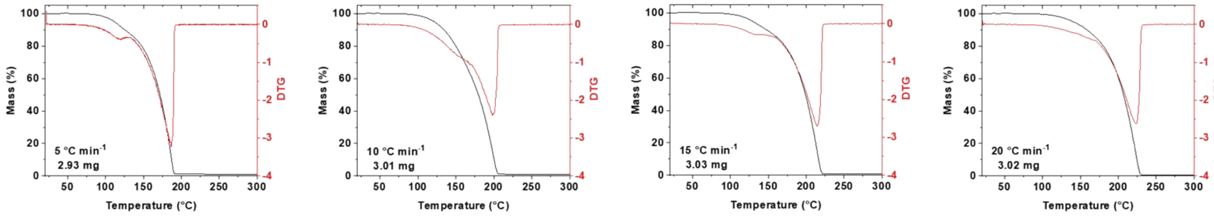


**Figure S16.** Thermogravimetric analysis of $WH_2(iPrCp)_2$. TG traces at nominally 3 mg and variable temperature ramp rates with derivative thermogravimetric (DTG) traces shown.

**Table S8.** Measured and calculated thermophysical parameters from TGA of $WH_2(iPrCp)_2$ at nominally 3 mg and variable temperature ramp rates.

| Heating rate (°C/min) | 5 | 10 | 15 | 20 |
|---|---|---|---|---|
| Sample mass (mg) | 2.93 | 3.01 | 3.03 | 3.02 |
| Slope (Arrhenius plot) | -6.17 | -4.57 | -6.17 | -6.67 |
| $R^2$ (Arrhenius plot) | 0.94 | 0.99 | 0.96 | 0.99 |
| $E_a$ (J/mol) | 51.30 | 38.01 | 51.28 | 55.45 |
| $R^2$ (Clausius-Clapeyron plot) | 0.94 | 0.99 | 0.97 | 0.99 |
| $\Delta H_{sub}$ (kJ/mol) | 53.05 | 39.82 | 53.13 | 57.33 |
| $T$ at 1 Torr (°C) | 136.70 | 109.98 | 127.58 | 128.19 |
| $T$ window (°C) | 112-189 | 121-203 | 129-218 | 141-227 |
| Residual mass (%) | 1.07 | 0.80 | 0.85 | 0.66 |

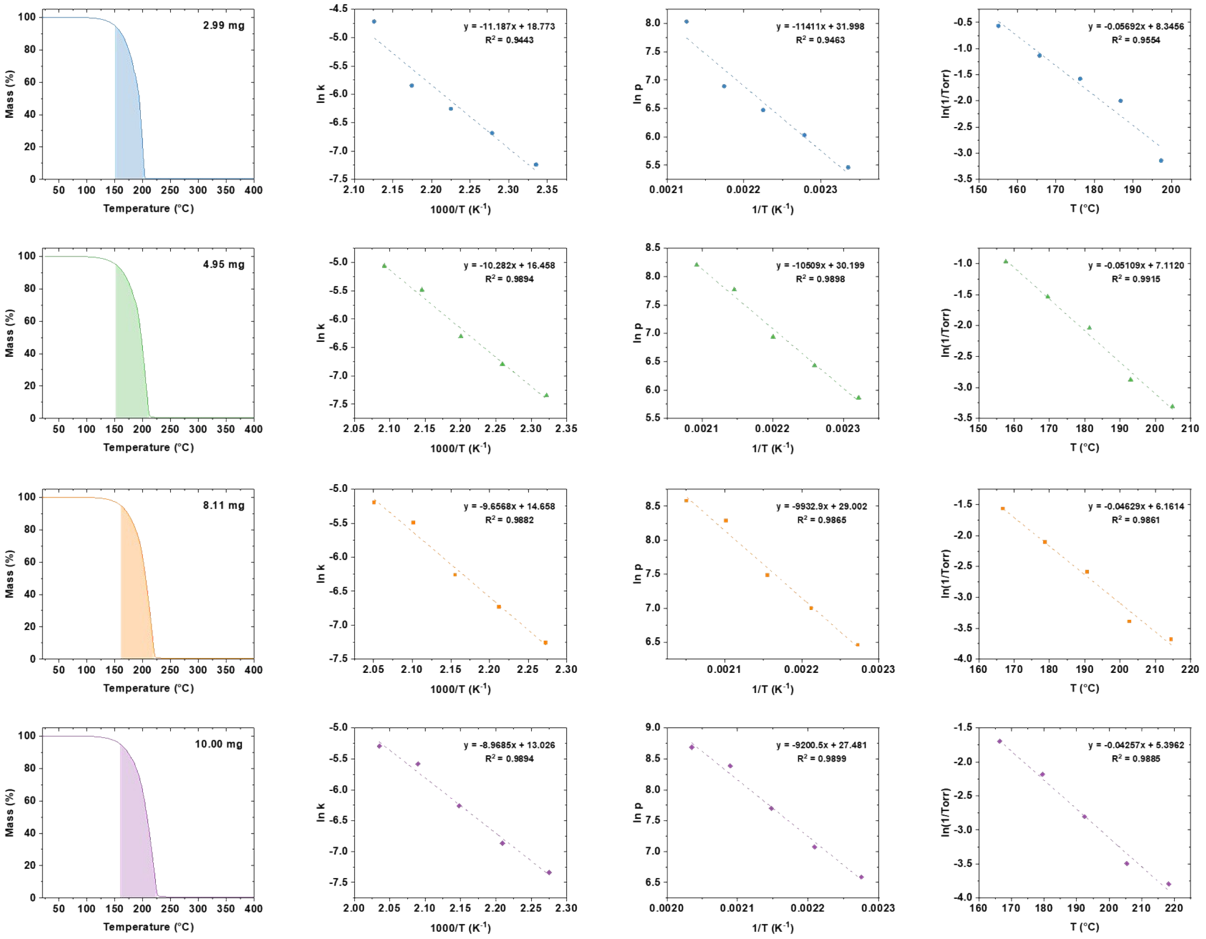


**Figure S17.** Thermogravimetric analysis of $WH_2(Cp)_2$. TG traces at constant temperature ramp rate of 10 °C/min and nominally 3 (blue), 5 (green), 8 (orange), and 10 (purple) mg (with actual masses displayed on graphs). Shaded regions underneath TG traces represent TGA temperature windows. Plots of ln *k* vs 1000/*T*, ln *p* vs 1/*T*, and ln(1/Torr) vs *T* used to derive $E_a$, $\Delta H_{sub}$, and *T* at 1 Torr, respectively. Equation of line of best fit and $R^2$ value displayed on each of these plots.

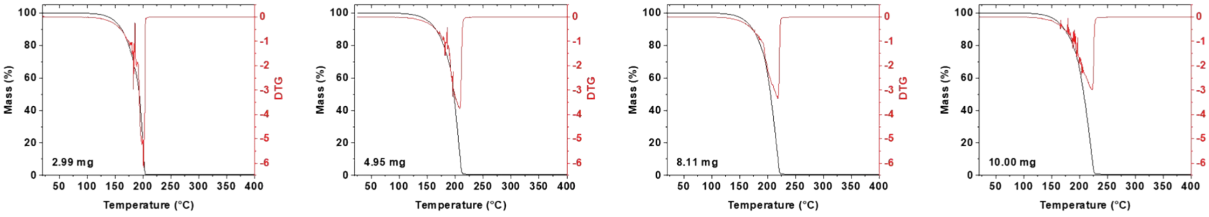


**Figure S18.** Thermogravimetric analysis of $WH_2(Cp)_2$. TG traces at constant temperature ramp rate of 10 °C/min and variable masses with derivative thermogravimetric (DTG) traces shown.

**Table S9.** Measured and calculated thermophysical parameters from TGA of $WH_2(Cp)_2$ at constant temperature ramp rate of 10 °C/min and variable masses.

| Sample mass (mg) | 2.99 | 4.95 | 8.11 | 10.00 |
|---|---|---|---|---|
| Slope (Arrhenius plot) | -11.19 | -10.28 | -9.66 | -8.97 |
| $R^2$ (Arrhenius plot) | 0.94 | 0.99 | 0.99 | 0.99 |
| $E_a$ (J/mol) | 93.01 | 85.48 | 80.29 | 74.56 |
| $R^2$ (Clausius-Clapeyron plot) | 0.95 | 0.99 | 0.99 | 0.99 |
| $\Delta H_{sub}$ (kJ/mol) | 94.88 | 87.38 | 82.59 | 76.50 |
| $T$ at 1 Torr (°C) | 146.62 | 139.21 | 133.10 | 126.76 |
| $T$ window (°C) | 150-203 | 152-210 | 161-220 | 160-225 |
| Residual mass (%) | 0.58 | 0.49 | 0.59 | 0.57 |

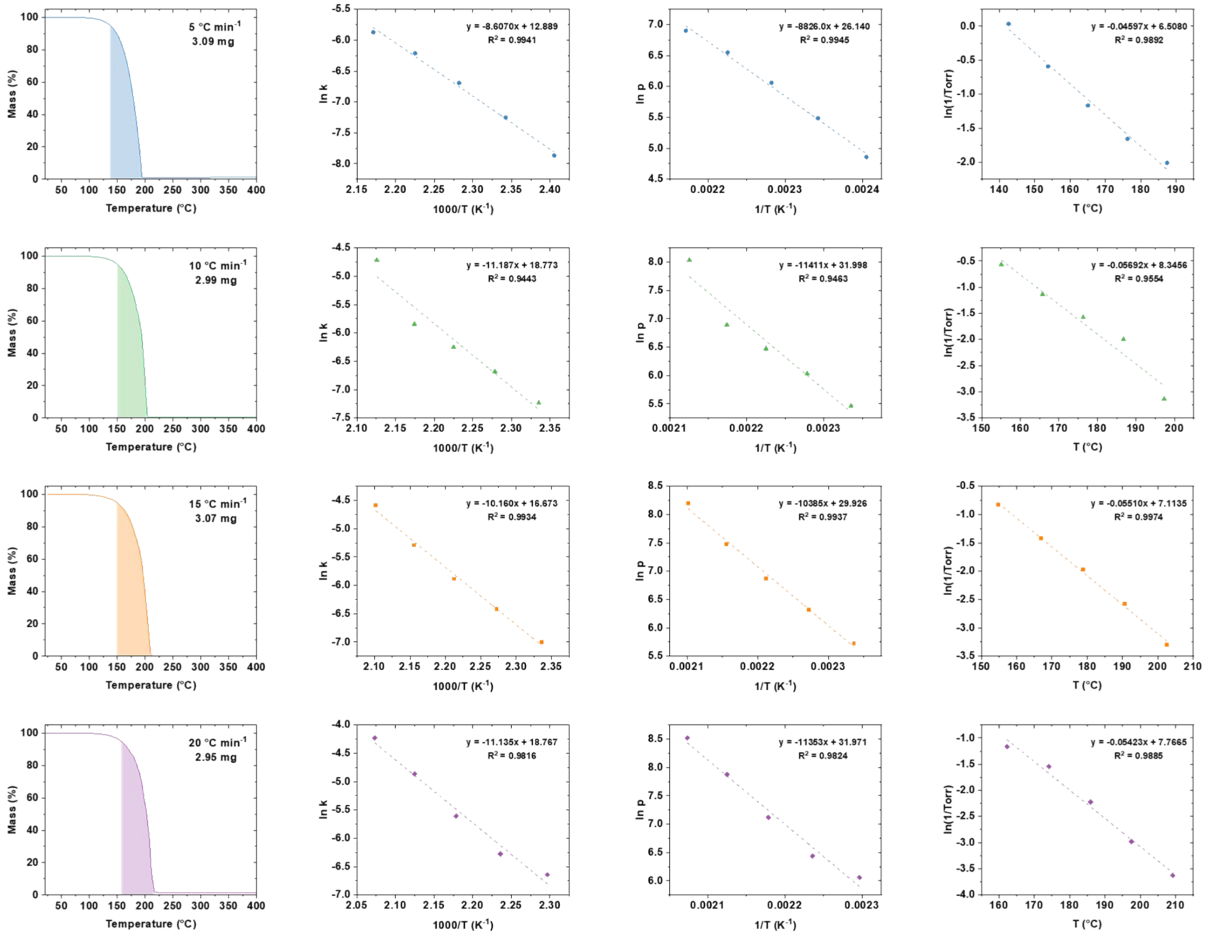


**Figure S19.** Thermogravimetric analysis of $WH_2(Cp)_2$. TG traces at nominally 3 mg and variable temperature ramp rates of 5 (blue), 10 (green), 15 (orange), and 20 (purple) °C/min (with actual masses displayed on graphs). Shaded regions underneath TG traces represent TGA temperature windows. Plots of ln $k$ vs 1000/$T$, ln $p$ vs 1/$T$, and ln(1/Torr) vs $T$ used to derive $E_a$, $\Delta H_{sub}$, and $T$ at 1 Torr, respectively. Equation of line of best fit and $R^2$ value displayed on each of these plots.

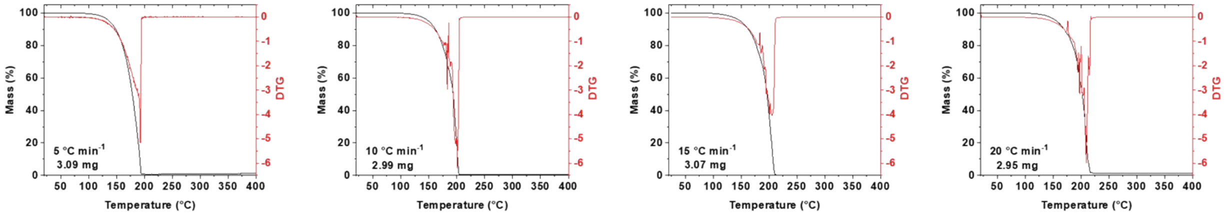


**Figure S20.** Thermogravimetric analysis of $WH_2(Cp)_2$. TG traces at nominally 3 mg and variable temperature ramp rates with derivative thermogravimetric (DTG) traces shown.

**Table S10.** Measured and calculated thermophysical parameters from TGA of $WH_2(Cp)_2$ at nominally 3 mg and variable temperature ramp rates.

| Heating rate (°C/min) | 5 | 10 | 15 | 20 |
|---|---|---|---|---|
| Sample mass (mg) | 3.09 | 2.99 | 3.07 | 2.95 |
| Slope (Arrhenius plot) | -8.61 | -11.19 | -10.16 | -11.14 |
| $R^2$ (Arrhenius plot) | 0.99 | 0.94 | 0.99 | 0.98 |
| $E_a$ (J/mol) | 71.56 | 93.01 | 84.47 | 92.58 |
| $R^2$ (Clausius-Clapeyron plot) | 0.99 | 0.95 | 0.99 | 0.98 |
| $\Delta H_{sub}$ (kJ/mol) | 73.38 | 94.88 | 86.35 | 94.39 |
| $T$ at 1 Torr (°C) | 141.57 | 146.62 | 129.10 | 143.21 |
| $T$ window (°C) | 137-193 | 150-203 | 149-208 | 157-215 |
| Residual mass (%) | 1.25 | 0.58 | 0.00 | 1.43 |

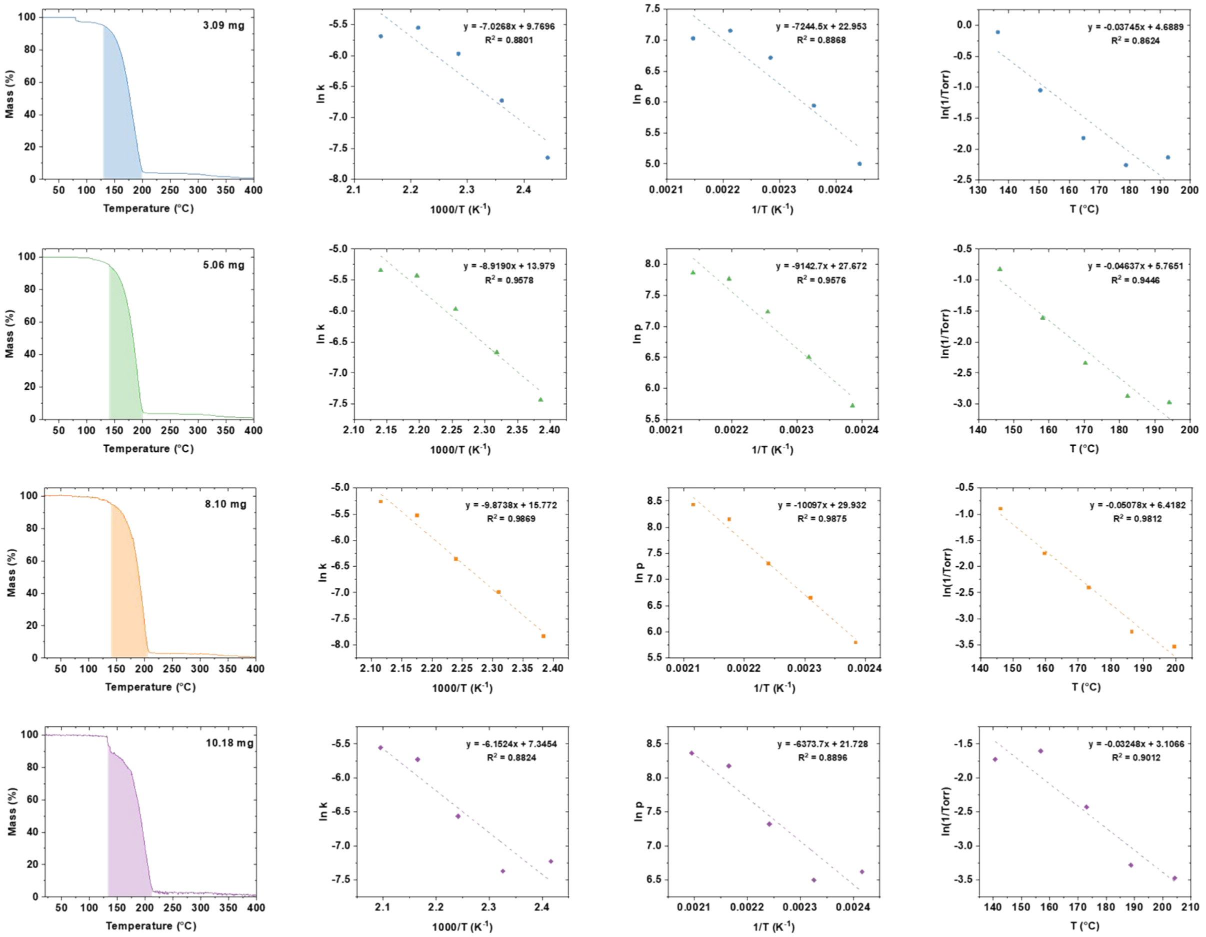


**Figure S21.** Thermogravimetric analysis of $[WCl_5]_2$. TG traces at constant temperature ramp rate of 10 °C/min and nominally 3 (blue), 5 (green), 8 (orange), and 10 (purple) mg (with actual masses displayed on graphs). Shaded regions underneath TG traces represent TGA temperature windows. Plots of ln *k* vs 1000/*T*, ln *p* vs 1/*T*, and ln(1/Torr) vs *T* used to derive $E_a$, $\Delta H_{sub}$, and *T* at 1 Torr, respectively. Equation of line of best fit and $R^2$ value displayed on each of these plots.

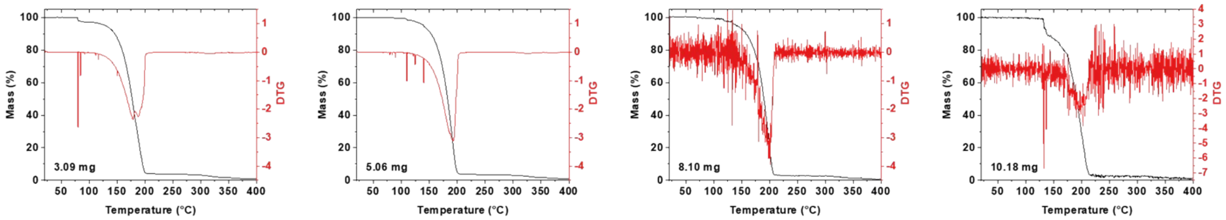


**Figure S22.** Thermogravimetric analysis of $[WCl_5]_2$. TG traces at constant temperature ramp rate of 10 °C/min and variable masses with derivative thermogravimetric (DTG) traces shown.

**Table S11.** Measured and calculated thermophysical parameters from TGA of $[WCl_5]_2$ at constant temperature ramp rate of 10 °C/min and variable masses.

| Sample mass (mg) | 3.09 | 5.06 | 8.10 | 10.18 |
|---|---|---|---|---|
| Slope (Arrhenius plot) | -7.03 | -8.92 | -9.87 | -6.15 |
| $R^2$ (Arrhenius plot) | 0.88 | 0.96 | 0.99 | 0.88 |
| $E_a$ (J/mol) | 58.42 | 74.15 | 82.09 | 51.15 |
| $R^2$ (Clausius-Clapeyron plot) | 0.89 | 0.96 | 0.99 | 0.89 |
| $\Delta H_{sub}$ (kJ/mol) | 60.23 | 76.02 | 83.95 | 52.99 |
| $T$ at 1 Torr (°C) | 125.20 | 124.33 | 126.39 | 95.65 |
| $T$ window (°C) | 130-200 | 140-200 | 140-206 | 133-212 |
| Residual mass (%) | 0.81 | 1.03 | 0.71 | 1.29 |

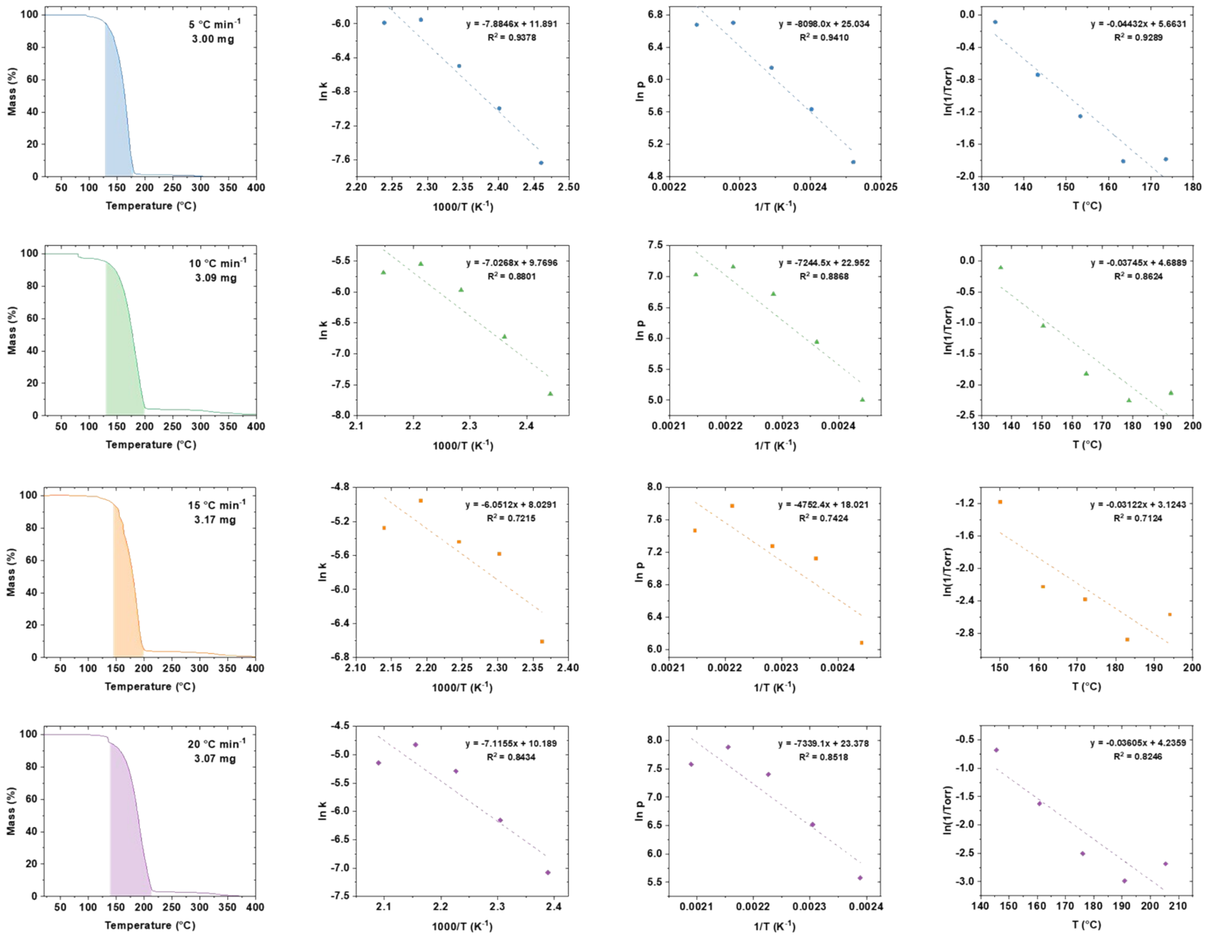


**Figure S23.** Thermogravimetric analysis of $[WCl_5]_2$. TG traces at nominally 3 mg and variable temperature ramp rates of 5 (blue), 10 (green), 15 (orange), and 20 (purple) °C/min (with actual masses displayed on graphs). Shaded regions underneath TG traces represent TGA temperature windows. Plots of ln *k* vs 1000/*T*, ln *p* vs 1/*T*, and ln(1/Torr) vs *T* used to derive $E_a$, $\Delta H_{sub}$, and *T* at 1 Torr, respectively. Equation of line of best fit and $R^2$ value displayed on each of these plots.

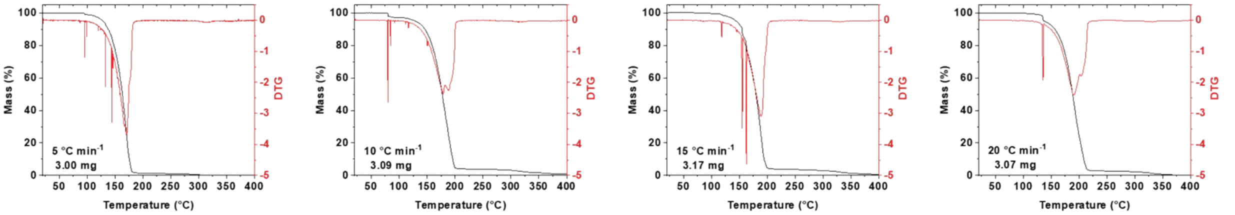


**Figure S24.** Thermogravimetric analysis of $[WCl_5]_2$. TG traces at nominally 3 mg and variable temperature ramp rates with derivative thermogravimetric (DTG) traces shown.

**Table S12.** Measured and calculated thermophysical parameters from TGA of $[WCl_5]_2$ at nominally 3 mg and variable temperature ramp rates.

| Heating rate (°C/min) | 5 | 10 | 15 | 20 |
|---|---|---|---|---|
| Sample mass (mg) | 3.00 | 3.09 | 3.17 | 3.07 |
| Slope (Arrhenius plot) | -7.88 | -7.03 | -6.05 | -7.12 |
| $R^2$ (Arrhenius plot) | 0.94 | 0.88 | 0.72 | 0.84 |
| $E_a$ (J/mol) | 65.55 | 58.42 | 50.31 | 59.16 |
| $R^2$ (Clausius-Clapeyron plot) | 0.94 | 0.89 | 0.74 | 0.85 |
| $\Delta H_{sub}$ (kJ/mol) | 67.33 | 60.23 | 39.51 | 61.02 |
| $T$ at 1 Torr (°C) | 127.78 | 125.20 | 100.07 | 117.50 |
| $T$ window (°C) | 128-178 | 130-200 | 145-199 | 138-213 |
| Residual mass (%) | 0.00 | 0.81 | 0.67 | 0.00 |

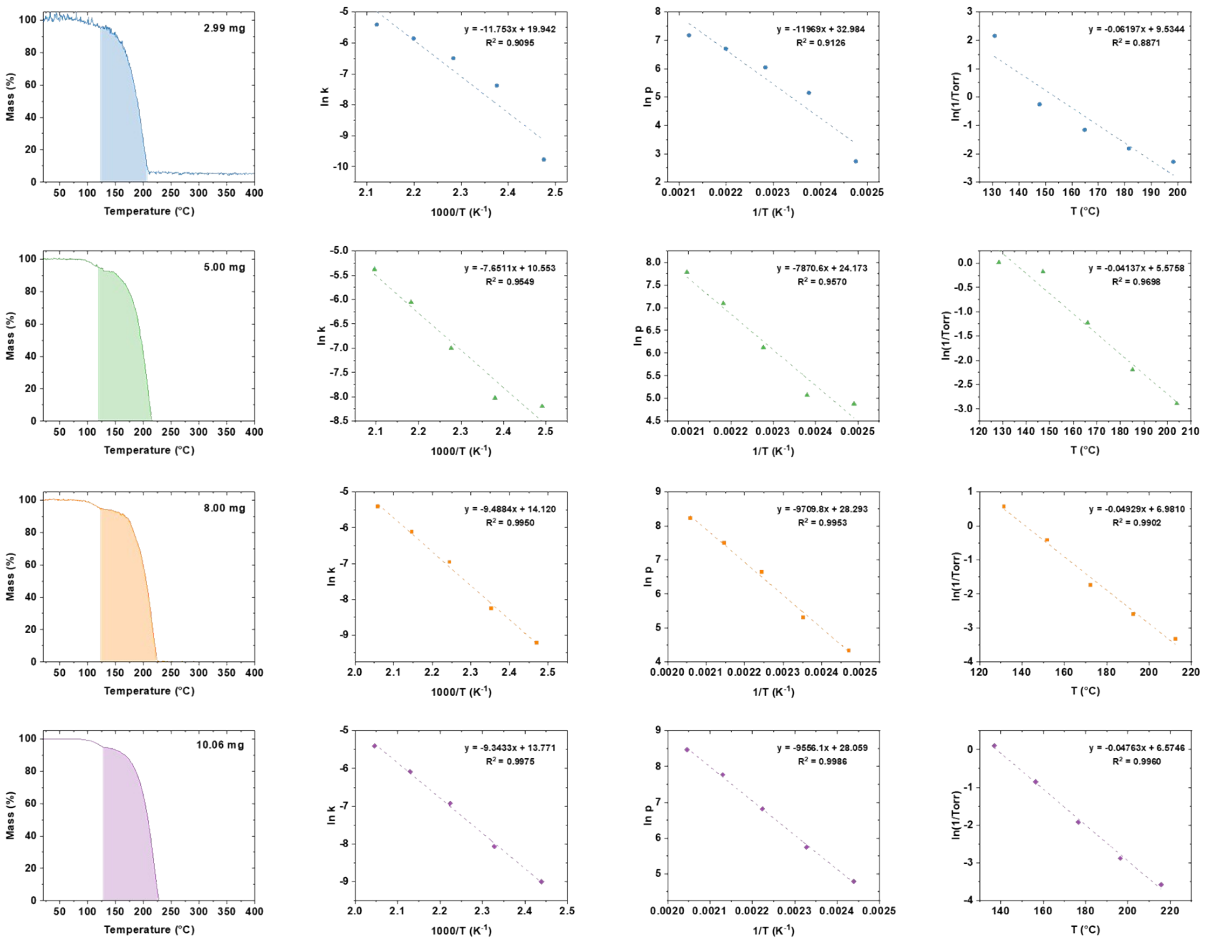


**Figure S25.** Thermogravimetric analysis of $WCl_6$. TG traces at constant temperature ramp rate of 10 °C/min and nominally 3 (blue), 5 (green), 8 (orange), and 10 (purple) mg (with actual masses displayed on graphs). Shaded regions underneath TG traces represent TGA temperature windows. Plots of ln *k* vs 1000/*T*, ln *p* vs 1/*T*, and ln(1/Torr) vs *T* used to derive $E_a$, $\Delta H_{sub}$, and *T* at 1 Torr, respectively. Equation of line of best fit and $R^2$ value displayed on each of these plots.

**Figure S26.** Thermogravimetric analysis of $WCl_6$. TG traces at constant temperature ramp rate of 10 °C/min and variable masses with derivative thermogravimetric (DTG) traces shown.

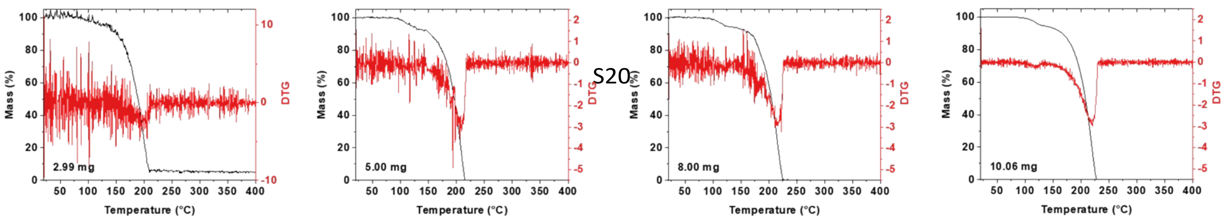

**Table S13.** Measured and calculated thermophysical parameters from TGA of $WCl_6$ at constant temperature ramp rate of 10 °C/min and variable masses.

| Sample mass (mg) | 2.99 | 5.00 | 8.00 | 10.06 |
|---|---|---|---|---|
| Slope (Arrhenius plot) | -11.75 | -7.65 | -9.49 | -9.34 |
| $R^2$ (Arrhenius plot) | 0.91 | 0.95 | 1.00 | 1.00 |
| $E_a$ (J/mol) | 97.71 | 63.61 | 78.89 | 77.68 |
| $R^2$ (Clausius-Clapeyron plot) | 0.91 | 0.96 | 1.00 | 1.00 |
| $\Delta H_{sub}$ (kJ/mol) | 99.52 | 65.44 | 80.73 | 79.45 |
| $T$ at 1 Torr (°C) | 153.86 | 134.78 | 141.63 | 138.03 |
| $T$ window (°C) | 122-207 | 119-213 | 122-222 | 127-225 |
| Residual mass (%) | 5.00 | 0.00 | 0.00 | 0.00 |

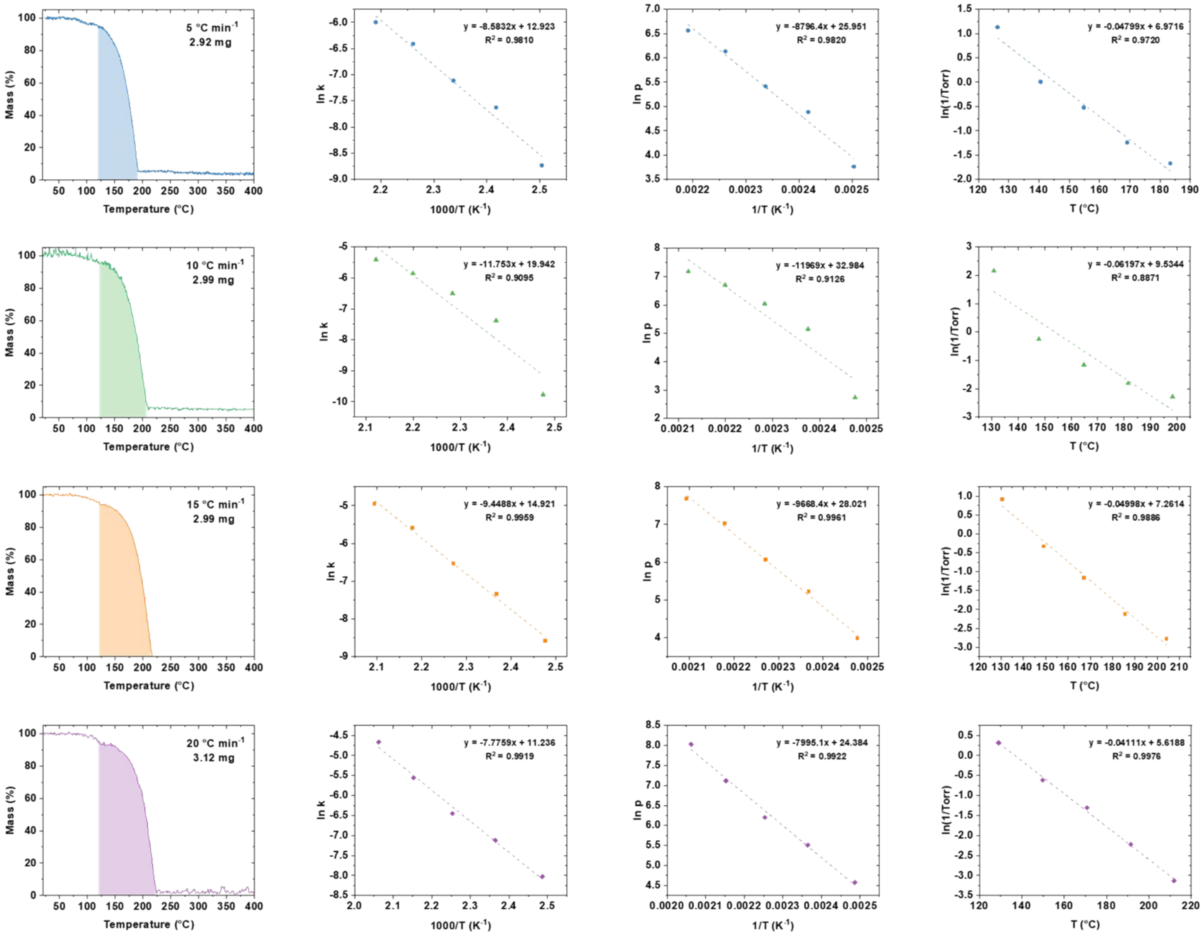


**Figure S27.** Thermogravimetric analysis of $WCl_6$. TG traces at nominally 3 mg and variable temperature ramp rates of 5 (blue), 10 (green), 15 (orange), and 20 (purple) °C/min (with actual masses displayed on graphs). Shaded regions underneath TG traces represent TGA temperature windows. Plots of ln $k$ vs 1000/$T$, ln $p$ vs 1/$T$, and ln(1/Torr) vs $T$ used to derive $E_a$, $\Delta H_{sub}$, and $T$ at 1 Torr, respectively. Equation of line of best fit and $R^2$ value displayed on each of these plots.

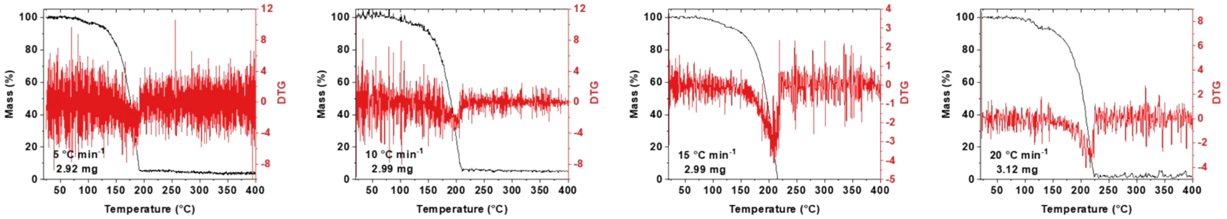


**Figure S28.** Thermogravimetric analysis of $WCl_6$. TG traces at nominally 3 mg and variable temperature ramp rates with derivative thermogravimetric (DTG) traces shown.

**Table S14.** Measured and calculated thermophysical parameters from TGA of $WCl_6$ at nominally 3 mg and variable temperature ramp rates.

| Heating rate (°C/min) | 5 | 10 | 15 | 20 |
|---|---|---|---|---|
| Sample mass (mg) | 2.92 | 2.99 | 2.99 | 3.12 |
| Slope (Arrhenius plot) | -8.59 | -11.75 | -9.45 | -7.78 |
| $R^2$ (Arrhenius plot) | 0.98 | 0.91 | 1.00 | 0.99 |
| $E_a$ (J/mol) | 71.44 | 97.71 | 78.56 | 64.65 |
| $R^2$ (Clausius-Clapeyron plot) | 0.98 | 0.91 | 1.00 | 0.99 |
| $\Delta H_{sub}$ (kJ/mol) | 73.14 | 99.52 | 80.39 | 66.48 |
| $T$ at 1 Torr (°C) | 145.27 | 153.86 | 145.29 | 136.68 |
| $T$ window (°C) | 119-190 | 122-207 | 121-214 | 119-222 |
| Residual mass (%) | 3.52 | 5.00 | 0.00 | 1.16 |

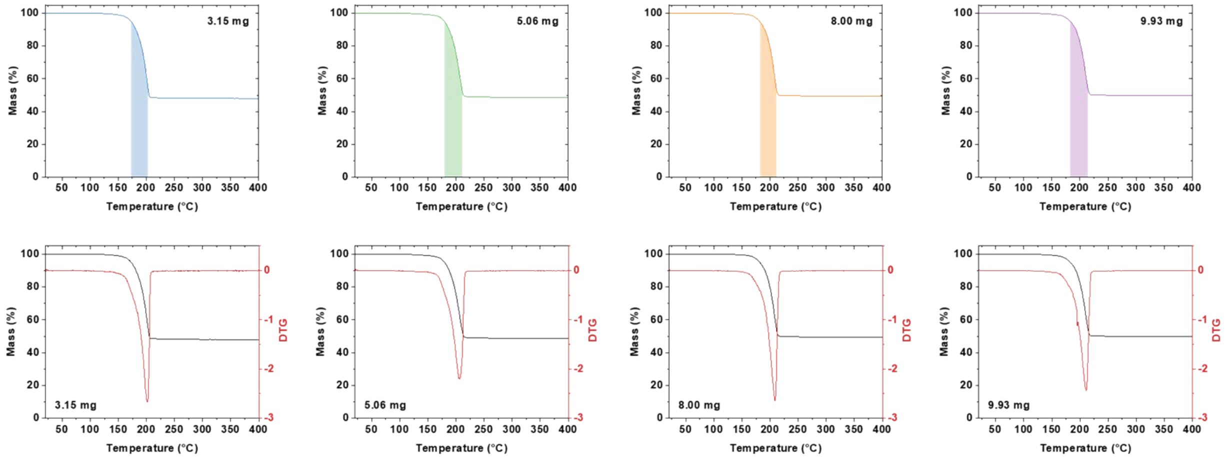


**Figure S29.** Thermogravimetric analysis of (mes)W(CO)$_3$. TG traces at constant temperature ramp rate of 10 °C/min and nominally 3 (blue), 5 (green), 8 (orange), and 10 (purple) mg (with actual masses displayed on graphs). Shaded regions underneath TG traces represent TGA temperature windows. Derivative thermogravimetric (DTG) traces also shown. Note (mes)W(CO)$_3$ led predominantly to decomposition products with 48.9 ± 0.8 % average residual mass precluding extraction of sublimation kinetics.

**Table S15.** Measured and calculated thermophysical parameters from TGA of (mes)WCO)$_3$ at constant temperature ramp rate of 10 °C/min and variable masses.

| Sample mass (mg) | 3.15 | 5.06 | 8.00 | 9.93 |
|---|---|---|---|---|
| *T* window (°C) | 173-203 | 180-211 | 182-211 | 183-214 |
| Residual mass (%) | 48.00 | 48.57 | 49.43 | 49.77 |

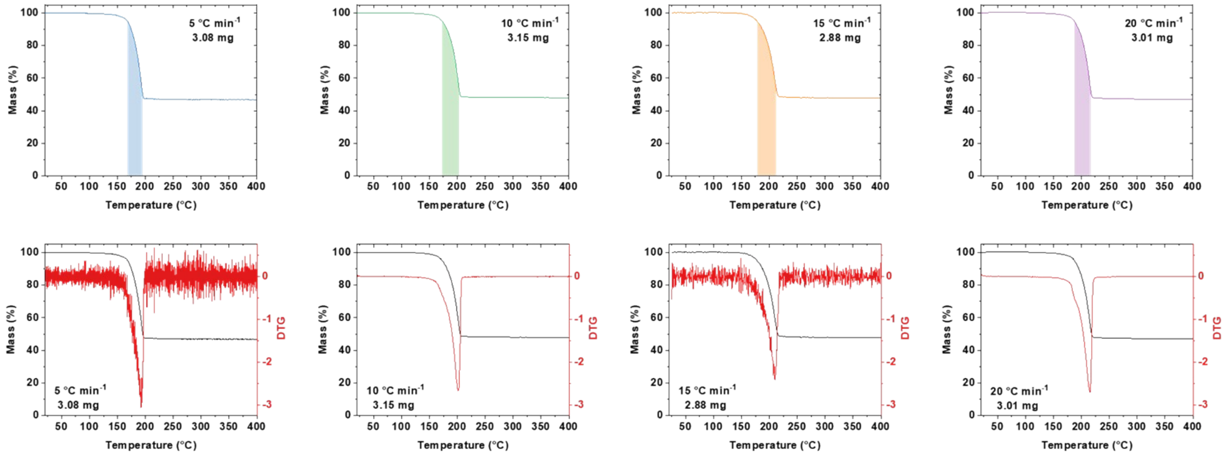


**Figure S30.** Thermogravimetric analysis of (mes)W(CO)$_3$. TG traces at nominally 3 mg and variable temperature ramp rates of 5 (blue), 10 (green), 15 (orange), and 20 (purple) °C/min (with actual masses displayed on graphs). Shaded regions underneath TG traces represent TGA temperature windows. Derivative thermogravimetric (DTG) traces also shown. Note (mes)W(CO)$_3$ led predominantly to decomposition products with 47.5 ± 0.6 % average residual mass precluding extraction of sublimation kinetics.

**Table S16.** Measured and calculated thermophysical parameters from TGA of (mes)W(CO)$_3$ at nominally 3 mg and variable temperature ramp rates.

| Heating rate (°C/min) | 5 | 10 | 15 | 20 |
|---|---|---|---|---|
| Sample mass (mg) | 3.08 | 3.15 | 2.88 | 3.01 |
| *T* window (°C) | 168-195 | 173-203 | 178-213 | 188-217 |
| Residual mass (%) | 46.82 | 48.00 | 47.87 | 47.16 |

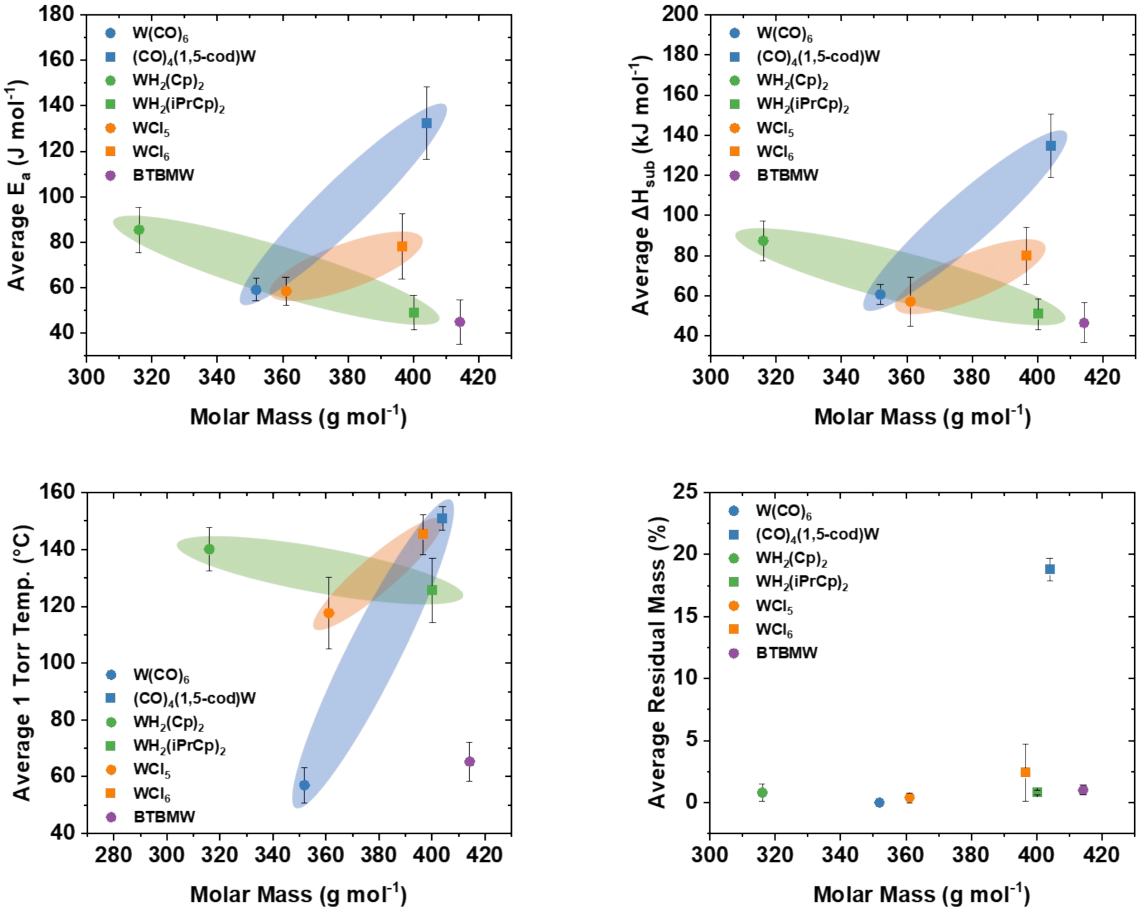


**Figure S31.** Plots comparing TGA-derived $E_a$, $\Delta H_{sub}$, $T$ at 1 Torr, and residual mass, respectively, of $W(CO)_6$, (1,5-cod)$W(CO)_4$, $WH_2Cp_2$, $WH_2$(iPrCp)$_2$, $WCl_6$, $[WCl_5]_2$, and BTBMW plotted against their molar masses. Thermophysical parameters are averages with error bars representing standard deviations, and based on TGA experiments in which sample loading was fixed at nominally 3 mg and temperature ramp rates were varied between 5, 10, 15, and 20 °C/min. The shaded regions group precursors together based on their ligand environments: carbonyl ligands (blue), cyclopentadienyl ligands (green), chloride ligands (orange), and amido/imido ligands (purple). Both $WH_2$(iPrCp)$_2$ and BTBMW are liquids at RT; the rest of the precursors shown are solids. Note (mes)$W(CO)_3$ led predominantly to decomposition products with 47.5 ± 0.6 % average residual mass precluding extraction of sublimation kinetics.

**Table S17.** TGA-derived $E_a$, $\Delta H_{sub}$ and 1 Torr temperature values from trials varying temperature ramp rate, with fixed 3 mg sample loading.[a]

| Molecule | $E_a$ (J/mol) | $\Delta H_{sub}$ (kJ/mol) | $T$ at 1 Torr (°C) | Res. Mass (%) |
|---|---|---|---|---|
| $W(CO)_6$ | 59.1 ± 4.9 | 60.6 ± 4.9 | 57.0 ± 6.3 | 0.0 ± 0.0 |
| (1,5-cod)$W(CO)_4$ | 132.4 ± 15.9 | 134.6 ± 15.8 | 150.9 ± 4.1 | 18.8 ± 0.9 |
| $WH_2Cp_2$ | 85.4 ± 10.0 | 87.3 ± 10.0 | 140.1 ± 7.7 | 0.8 ± 0.7 |
| $WH_2$(iPrCp)$_2$ | 49.0 ± 7.6 | 50.8 ± 7.6 | 125.6 ± 11.2 | 0.8 ± 0.2 |
| BTBMW | 44.9 ± 9.8 | 46.4 ± 9.9 | 65.3 ± 6.8 | 1.0 ± 0.4 |
| $WCl_6$ | 78.1 ± 14.3 | 79.9 ± 14.3 | 145.3 ± 7.0 | 2.4 ± 2.3 |
| $[WCl_5]_2$ | 58.4 ± 6.2 | 57.0 ± 12.1 | 117.6 ± 12.5 | 0.4 ± 0.4 |

[a] Note (mes)$W(CO)_3$ led predominantly to decomposition products with 47.5 ± 0.6 % average residual mass precluding extraction of sublimation kinetics.
